# Efficacy and safety of novel multifunctional M10 CAR-T cells in HIV-1-infected patients: a phase I, multicenter, single-arm, open-label study

**DOI:** 10.1038/s41421-024-00658-z

**Published:** 2024-05-14

**Authors:** Yunyu Mao, Qibin Liao, Youwei Zhu, Mingyuan Bi, Jun Zou, Nairong Zheng, Lingyan Zhu, Chen Zhao, Qing Liu, Li Liu, Jun Chen, Ling Gu, Zhuoqun Liu, Xinghao Pan, Ying Xue, Meiqi Feng, Tianlei Ying, Pingyu Zhou, Zhanshuai Wu, Jian Xiao, Renfang Zhang, Jing Leng, Yongtao Sun, Xiaoyan Zhang, Jianqing Xu

**Affiliations:** 1 Clinical Center of Biotherapy at Zhongshan Hospital & Institutes of Biomedical Sciences, Shanghai Institute of Infectious Disease and Biosecurity, Shanghai Public Health Clinical Center, Fudan University, Shanghai, China; 2 Department of Infectious Diseases, Tangdu Hospital, Air Force Medical University, Xi’an, Shaanxi China; 3 AIDS Clinical Treatment Center, The Fourth People’s Hospital of Nanning, Nanning, Guangxi China; 4 Shanghai Skin Disease Hospital, Tongji University, Shanghai, China; 5 Guangxi Key Laboratory of Translational Medicine for Treating High-Incidence Infectious Diseases with Integrative Medicine, Department of Medical Immunology, Guangxi University of Chinese Medicine, Nanning, Guangxi China

**Keywords:** Autoimmunity, Cell biology

## Abstract

Chimeric antigen receptor T (CAR-T) cells have been proposed for HIV-1 treatment but have not yet demonstrated desirable therapeutic efficacy. Here, we report newly developed anti-HIV-1 CAR-T cells armed with endogenic broadly neutralizing antibodies (bNAbs) and the follicle-homing receptor CXCR5, termed M10 cells. M10 cells were designed to exercise three-fold biological functions, including broad cytotoxic effects on HIV-infected cells, neutralization of cell-free viruses produced after latency reversal, and B-cell follicle homing. After demonstrating the three-fold biological activities, M10 cells were administered to treat 18 HIV-1 patients via a regimen of two allogenic M10 cell infusions with an interval of 30 days, with each M10 cell infusion followed by two chidamide stimulations for HIV-1 reservoir activation. Consequently, 74.3% of M10 cell infusions resulted in significant suppression of viral rebound, with viral loads declining by an average of 67.1%, and 10 patients showed persistently reduced cell-associated HIV-1 RNA levels (average decrease of 1.15 log10) over the 150-day observation period. M10 cells were also found to impose selective pressure on the latent viral reservoir. No significant treatment-related adverse effects were observed. Overall, our study supported the potential of M10 CAR-T cells as a novel, safe, and effective therapeutic option for the functional cure of HIV-1/AIDS.

## Introduction

Acquired immunodeficiency syndrome (AIDS) remains a major global threat to public health, with over 37.7 million people living with human immunodeficiency virus (HIV)-1 (PLWH) worldwide. The application of antiretroviral therapy (ART) has significantly improved the clinical outcome of PLWH. However, due to the persistence of the viral reservoir in blood and other body tissues, which ART cannot eradicate, PLWH always require lifelong medication, suffering from associated drug toxicities and facing the risk of drug resistance. Thus, finding a therapy to achieve ART-free remission represents the next step toward conquering HIV.

Chimeric antigen receptor T (CAR-T) cell-based therapy has recently emerged as a promising approach to treating HIV-1 infection patients by enhancing HIV-1-specific immune responses. Anti-HIV-1 CAR-T cells are T cells engineered to specifically recognize HIV-1 envelope glycoprotein (Env) expressed on the surface of virus-producing cells. Although the first generation of anti-HIV-1 CAR-T cells, CD4ζ CAR-T cells, showed only modest antiviral efficacy in phase I clinical trials^1,2^, researchers have improved designs of the CAR, e.g., decorating the CAR with multispecific antigen recognition domains and costimulatory signaling domains, and the resultant CAR-T cells demonstrated several advantages over the prototype, including enhanced expansion, persistence, and antiviral activities^3–7^. However, to date, none of the tested anti-HIV-1 CAR-T cells have succeeded in the long-term prevention of viral rebound following ART discontinuation. A possible explanation for this inefficacy is the emergence of escape variants, which has been observed in HIV patients receiving a single administration of monofunctional anti-HIV-1 CAR-T cells^8^. Thus, broadening CAR recognition to restrain virus escape and sharpening it to target viral reservoirs for elimination could be the key to developing superior anti-HIV-1 CAR-T cells for an HIV-1 cure.

Combining with broadly neutralizing antibodies (bNAbs) represents an intriguing strategy to enhance the functionality of anti-HIV-1 CAR-T cells. In contrast to the CAR, whose action relies on recognizing surface-expressed viral Env epitopes, bNAbs can neutralize cell-free viruses by directly blocking their binding to host cells and enhance HIV-1-specific immune responses by engaging Fc receptors. A synergistic anti-HIV effect was observed with the combined use of bNAbs and CD8^+^ T cells^9^. Moreover, in a recent study, HIV-1-specific T cells engineered to secrete bNAbs displayed superior activity in suppressing HIV-1 replication in vitro^10^. However, there has been no exploration of whether such a two-in-one approach could enhance treatment efficacy when applied to HIV patients. On the other hand, a critical determinant of the in vivo performance of anti-HIV-1 CAR-T cells is their tissue distribution, with a better efficacy arising from more effective and broader targeting of viral reservoirs. Among body compartments, B-cell follicles have been identified as the privileged sanctuary for both latent and active HIV-1 viruses during chronic HIV-1 infection^11,12^. Research has shown that failed accumulation of HIV-1-specific cytotoxic lymphocytes (CTLs) within lymphoid follicles underpins persistent viral replication^13^. The chemokine receptor CXCR5 is well known as a critical factor driving the migration of lymphocytes into B-cell follicles^14–17^. In line with this directing role of CXCR5, CXCR5-expressing HIV-1-specific T cells were found to possess potent antiviral activities in nonhuman primate models^15,18^, implicating B-cell follicle targeting as a promising strategy to develop more effective anti-HIV CAR-T therapy.

In this study, we propose to combine the aforementioned promising strategies into a single therapeutic product, tri-functional CAR-T cells termed M10. M10 cells feature a bispecific CAR molecule to exert Env-specific cytotoxic effects, a 10E8-derived single-chain variable fragment-Fc (scFv-Fc) fusion antibody to neutralize cell-free viruses, and the follicle-homing receptor CXCR5 to promote cellular migration into the germinal center to target viral reservoirs. After validating the intended properties of M10 cells in in vitro assays, we examined their efficacy in 18 PLWH using a treatment scheme combining HIV-1 reactivation with M10 cell infusion. Accordingly, we observed a marked reduction in both plasma viremia titers and cell-associated HIV-1 RNA (CA HIV-1 RNA) levels after M10 cell infusion. These data support the adoptive transfer of M10 CAR-T cells as a novel tool for developing a functional cure for HIV-1 infection.

## Results

### Design and production of M10 CAR-T cells

**Fig. 1:**
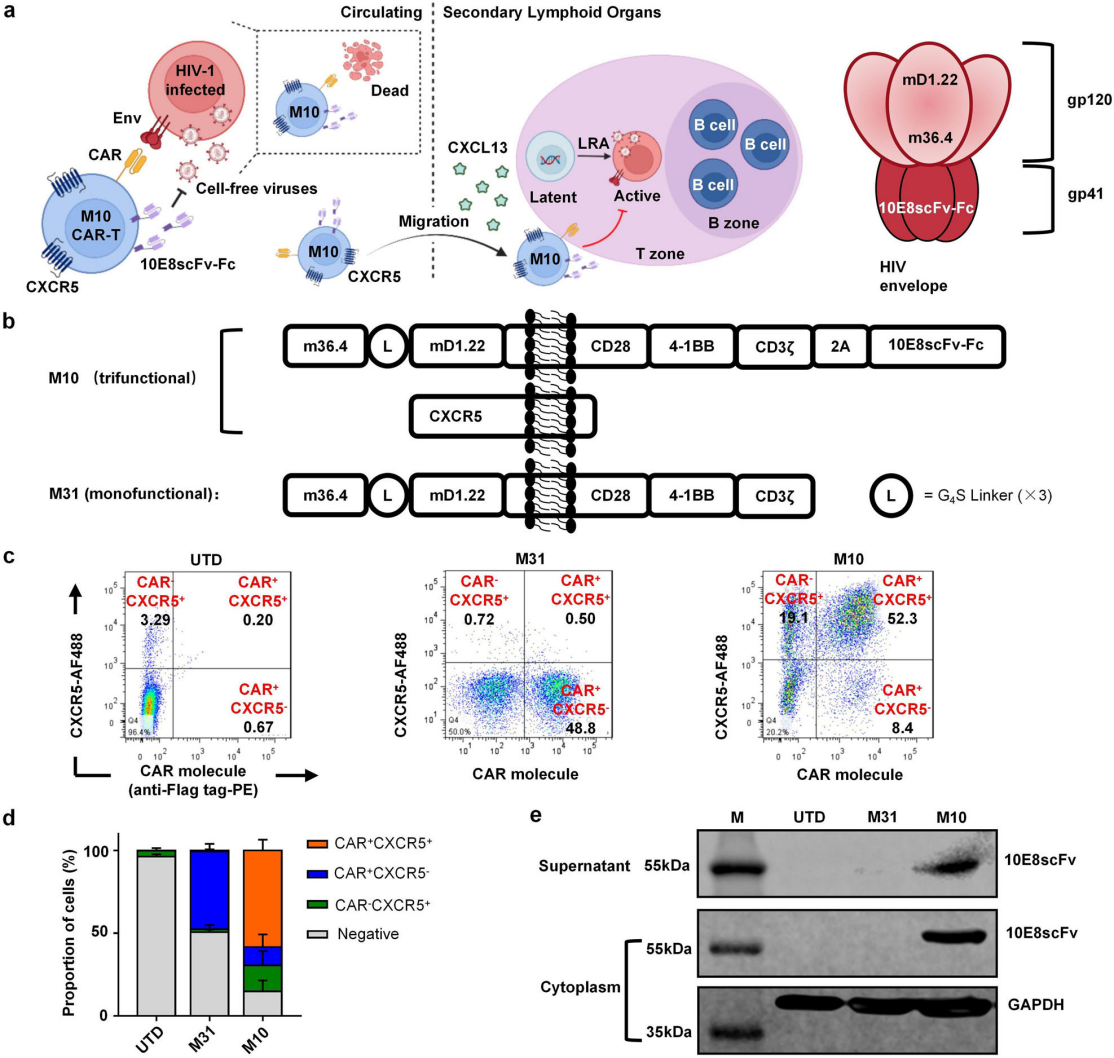
Design and production of M10 CAR-T cells. **a** Cartoon illustration of the triple biological functions possessed by M10 CAR-T cells, including cytotoxic effects against Env positive target cells, neutralization of cell-free virions, and B-cell follicle homing capacity. **b** Gene structure of M10 encoded by two lentivirus vectors. M31 served as a monofunctional CAR-T control. **c**, **d** Representative detection of CAR molecule (via Flag tag) and CXCR5 on the surface of transduced primary T cells using flow cytometry (*n* = 3 donors). **e** The expression of 10E8scFv-Fc was verified by western blotting analysis using goat anti-human IgG antibody.

M10 CAR-T cells were designed to exercise three-fold biological functions, including broad cytotoxic effects on HIV-infected cells conferred by cross-clade recognition of Env expressed on the cell surface, neutralization of cell-free viruses produced after latency reversal, and B-cell follicle homing (Fig. 1a). Previous research reported the design of a bispecific fusion protein consisting of m36.4, a single-domain antibody targeting the CD4i coreceptor-binding site, and mD1.22, a mutant of the CD4 D1 domain targeting the CD4-binding site, which, by recognizing the HIV-1 Env protein with high specificity and affinity, demonstrated broad and potent neutralizing activity^4,19^. Considering the advantage of such a design for effective Env engagement, we generated an M31 CAR molecule (designated “3” for m36.4 and “1” for mD1.22) by fusing m36.4 to mD1.22 via a 3× G_4_S linker, followed by a CD28 hinge and transmembrane domain, CD28 and 4-1BB costimulatory domain, and CD3ζ signaling domain (Fig. 1b). To endow CAR-T cells with the function of cell-free virus clearance, we constructed 10E8scFv-Fc, an Fc-fusion version of 10E8, which is a broad and potent bNAb whose binding site does not overlap with the CAR molecule, by ligating 10E8 to a mutant of IgG1 Fc capable of mediating antibody-dependent cellular cytotoxicity (ADCC). To introduce the CAR moiety 10E8scFv-Fc and CXCR5 into T cells, we generated two HIV-1-based lentivirus vectors, as shown in Fig. 1b, with the CAR moiety and 10E8scFv-Fc encoded in one and CXCR5 in the other. M31 served as a conventional monofunctional CAR-T cell control in the following experiments.

To test whether the three functionality modules can be efficiently delivered and expressed simultaneously, we transduced primary T cells with both lentiviruses and evaluated CAR and CXCR5 expression by flow cytometry. As shown in Fig. 1c, d, after a single round of transduction, CAR^+^CXCR5^+^ double-positive cells accounted for ~58% of M10 CAR-T cells, which also included a minority of CAR^+^ or CXCR5^+^ single-positive cells. The expression of the 10E8scFv-Fc antibody was verified by western blotting analysis, as shown in Fig. 1e.

### In vitro characterization of M10 CAR-T cells confirmed their multifunctionalities

**Fig. 2:**
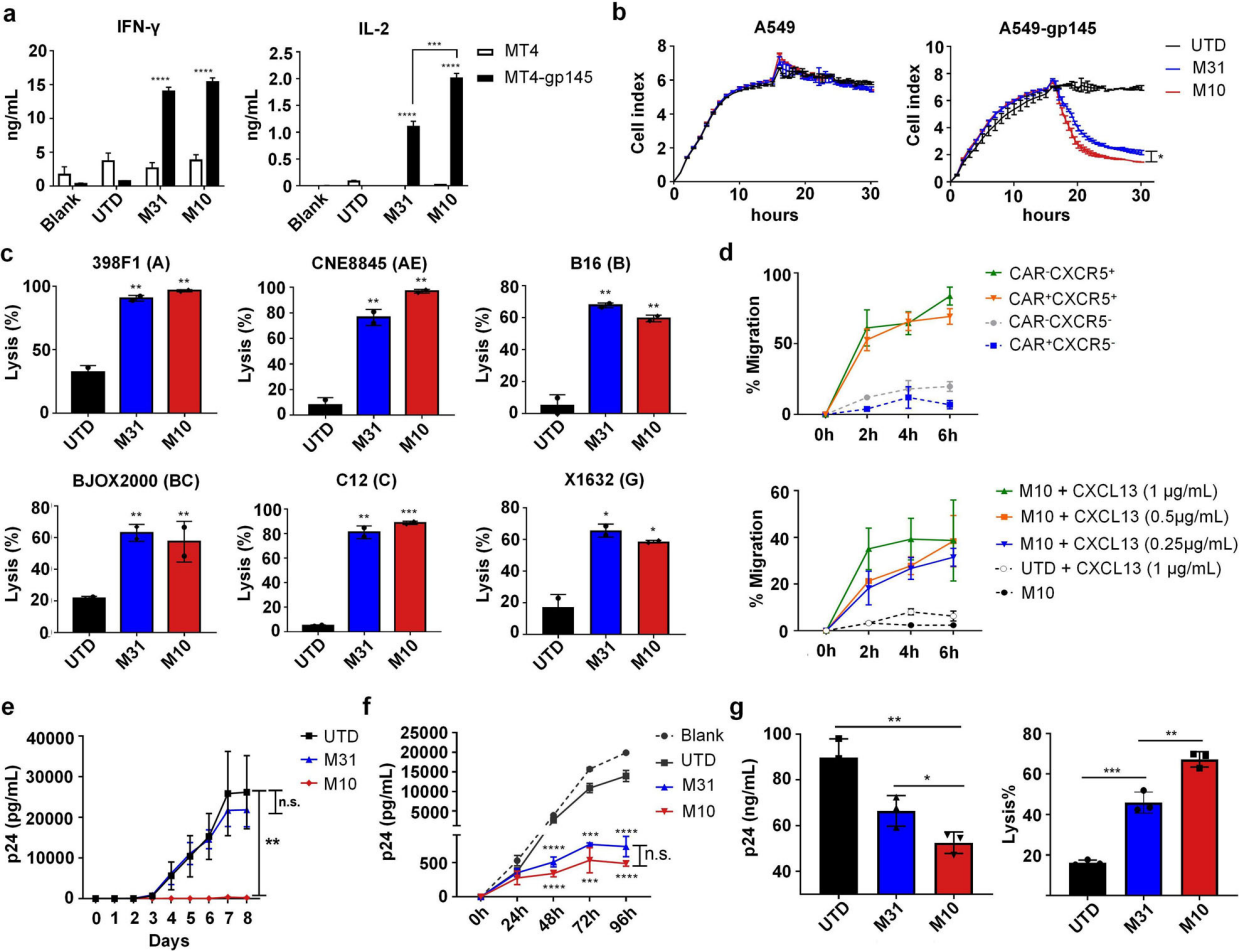
M10 CAR-T cells exerted strong cytotoxic effects, selective migration, and superior neutralization in vitro. **a** The levels of IFN-γ and IL-2 in the supernatant secreted by M10/M31 or UTD T cells in 24 h after antigen stimulation (E:T, 1:1) were measured by ELISA. **b** The cytotoxic effects against A549 cells expressing with/without gp145 (E:T, 1:1) was measured through RTCA. The measured relative change in electrical impedance is expressed as the Cell Index (CI), an integrative parameter whose magnitude depends on not only cell numbers but also other cell properties, including adhesive strength, viability, and morphology. **c** The cytotoxic effects against TZM-bl cells expressing different clade-derived Env variants (E:T, 1:1) were measured through RTCA. **d** Co-expression of CXCR5 promotes selective migration of primary T cells toward CXCL13 in vitro. The percentage of transduced T cells that migrated toward CXCL13 was measured in 24-well transwell plates. **e** Activated CD4^+^ T cells were challenged with the mixture of HIV-1 IIIB viruses and UTD/M31/M10 culture supernatant for 12 h and the concentration of HIV-1 p24 antigen in the supernatant was monitored by ELISA every 24 h. **f** Cytotoxicity effects against HIV-1-infected CD4^+^ T cells (E:T, 1:1). **g** Cytotoxicity effects against reactivated ACH2 cells (E:T, 1:1). The concentration of HIV-1 p24 antigen was measured after 72 h by ELISA. The specific cytotoxicity after PMA activation was measured through flow cytometry. Significance was analyzed using Student’s *t*-test (****P < 0.0001, ***P < 0.001, **P < 0.01, *P < 0.05, n.s. not significant).

We first characterized M10 CAR-T cells in vitro to examine whether they possess multifunctionality as designed. To verify whether the M31 CAR is functional in M10 CAR-T cells, we examined the target cell killing of M10 or M31 CAR-T cells using an effector-to-target (E:T) ratio of 1:1, with gp145-expressing or nonexpressing MT4 cells as target cells. As shown in Fig. 2a, coculturing with gp145^+^ target cells triggered robust secretion of IFN-γ and IL-2 for both M10 and M31 CAR-T cells but not control T cells (untransduced, UTD). In contrast, the presence of gp145^*−*^ target cells stimulated marginal cytokine production, demonstrating the target selectivity of the M31 CAR. We also observed a higher level of IL-2 secretion by activated M10 CAR-T cells than by their M31 counterparts. The in vitro cytotoxic potential of M10 cells was first evaluated using gp145^+^ A549 cells as the target cells, with wild-type A549 cells used as a negative control to assess the target selectivity. As shown in Fig. 2b, M10 CAR-T cells displayed more rapid cytotoxicity induction against gp145^+^ A549 cells than M31 CAR-T cells while showing no off-target killing of wild-type A549 cells (*P* = 0.025). The average killing efficacy of M10 cells at 6 h was 72.7%, which is higher than the 60.4% efficacy shown by M31 CAR-T cells. No cytotoxicity was observed for control UTD cells. We next evaluated whether M10 cells could recognize Env variants of different clades. To this end, TZM-bl cells were first infected with a lentivirus expressing different Env variants and then cocultured with CAR-T effector cells at an E:T ratio of 1:1, followed by monitoring the killing of TZM-bl cells by real-time cellular analysis (RTCA). As shown in Fig. 2c, both the M31 and M10 CAR mediated broad cytotoxic effects against target cells expressing Env from clade A (398F1), clade AE (CNE8845), clade B (B16), clade BC (BJOX2000), clade C (C12), or clade G (X1632). These data indicated that the M31 CAR moiety works as expected in the context of M10 CAR-T cells, enabling these cells to potently and selectively eliminate various Env^+^ targets in vitro.

We next evaluated whether CXCR5 expression promotes the selective migration of CAR-T cells, particularly M10 cells. To this end, we utilized an in vitro transwell migration assay. CAR^*−*^CXCR5^+^ and CAR^+^CXCR5^+^ T cells migrated toward CXCL13, the chemokine ligand for CXCR5, with similar efficiency, whereas CAR^+^CXCR5^*−*^ and CAR^*−*^CXCR5^*−*^ cells did not. Furthermore, M10 cells migration was dependent on the CXCL13 concentration (Fig. 2d). These results support the notion that expressing CXCR5 confers M10 cells the ability to migrate to a CXCL13-enriched environment, i.e., the germinal center, regarded as a crucial sanctuary of HIV-infected CD4^+^ T cells.

We also evaluated the 10E8scFv-Fc antibody-mediated functionality of M10 cells. Hence, we incubated CD4^+^ T cells with HIV-1 IIIB viruses in the presence of culture supernatants of control T cells or M10 cells for 12 h before measuring the concentration of HIV-1 p24 antigen in the supernatant every 24 h using ELISA. As shown in Fig. 2e, the supernatant concentration of p24 antigen in the M10 group was controlled at the baseline level throughout the 8-day observation period, consistent with M10 cells releasing sufficient 10E8scfv-Fc for effective neutralization of cell-free HIV viruses. In contrast, a continual increase in p24 levels was observed in the control T cell groups until Day 7 and remained high on Day 8. Furthermore, the M31 CAR moiety was shown not to mediate the infection of modified T cells by HIV-1 viruses (Supplementary Fig. S1).

To further evaluate the killing efficacy of M10 cells against productive HIV-1-infected cells, we used primary CD4^+^ T cells infected with HIV-1 IIIB viruses as target cells and daily measured p24 antigen levels in the supernatant after coculturing with M10/M31 or control T cells. As shown in Fig. 2f, the supernatant concentrations of p24 antigen were effectively controlled in the M10 group compared to those in the control groups. Given that latent HIV-1 represents the major challenge associated with ART treatment, we also examined the effect of M10 cells on the ACH2 cell line (a model for chronic HIV-1 infection) under conditions that imitate the reactivation of the latent viral reservoir. Coculturing with M10 cells resulted in a significant reduction in the level of p24 antigen in the supernatant of PMA-reactivated ACH2 cells to a greater degree than that after coculturing with M31 cells, confirming the ability of M10 cells to kill reactivated HIV-infected cells and their potential to reduce the viral reservoir during therapeutic use (Fig. 2g). Thus, our assessments revealed a superior antiviral capability of M10 over M31 cells, reflecting the advantage of arming anti-HIV-1 CAR-T cells with bNAbs to realize a dual antiretroviral mechanism. B-NDG mice bearing A549/gp145^+^A549 solid tumors were also used to evaluate the cytotoxic efficacy of M10 cells in vivo. We observed significant inhibition of tumor growth in those mice treated with M10 or M31 cells, suggesting that M10/M31 cells could selectively target and kill gp145^+^ cells in vivo (Supplementary Fig. S2).

### Infusion of allogeneic M10 cells was well tolerated in PLWH

With the above in vitro experiments and preclinical animal studies validating the anti-HIV efficacy of M10 cells, we sought to assess their therapeutic potential by conducting a multicenter, single-arm, phase I clinical trial between September 2020 and September 2022. Of the 18 participants enrolled in the study, 14 (77.8%) were males, and 4 (22.2%) were females. The median age was 31 years (range, 18–57 years), the median CD4 count at entry was 531 cells/μL (range, 301 to 1120 cells/μL), and the median duration from the initiation of ART to the entry date was 5.5 years (range, 0.5–12.8 years) (Table 1). All participants maintained virological suppression with plasma HIV-1 RNA < 20 copies/mL before enrollment and had no comorbidities or complications.

**Table 1:** Baseline clinical characteristics of enrolled participants.

ID	Age	Gender	Race	CD4 Count (at enrollment)	Years since first ART	ART at screening*	Plasma HIV-1 RNA(cp/mL)
001	24	Male	Asian	357	1.8	TDF + 3TC + EFV	< 20
002	37	Female	Asian	762	12.2	AZT + 3TC + NVP	< 20
003	27	Male	Asian	568	3.6	TDF + 3TC + EFV	< 20
004	38	Female	Asian	533	11.6	TAF + FTC + DTG	< 20
005	48	Male	Asian	477	11.3	TAF + FTC + DTG	< 20
006	24	Male	Asian	930	4.8	3TC + DTG	< 20
007	57	Male	Asian	461	7.7	TAF + FTC + DTG	< 20
008	36	Male	Asian	529	7.9	TDF + 3TC + LPV/r	< 20
009	32	Male	Asian	977	1.0	TDF + 3TC + EFV	< 20
010	30	Male	Asian	520	6.3	TDF + 3TC + EFV	< 20
011	38	Female	Asian	361	12.8	TDF + 3TC + NVP	< 20
012	23	Male	Asian	1069	6.4	TAF + FTC + DTG	< 20
013	18	Male	Asian	301	0.5	TDF + 3TC + EFV	< 20
014	30	Male	Asian	544	0.5	TDF + 3TC + EFV	< 20
015	28	Male	Asian	413	4.2	DTG + ABC + 3TC	< 20
016	40	Female	Asian	370	0.5	TDF + 3TC + EFV	< 20
017	38	Male	Asian	555	8.2	DTG + TAF + FTC	< 20
018	22	Male	Asian	1120	4.3	TDF + 3TC + EFV	< 20

^*^Abbreviations for antiretroviral therapies: *TDF* tenofovir disoproxil fumarate, *3TC* lamivudine, *EFV* efavirenz, *AZT* zidovudine, *NVP* nevirapine, *TAF* tenofovir alafenamide fumarate, *FTC* emtricitabine, *DTG* dolutegravir, *LPV/r* lopinavir/ritonavir, *ABC* abacavir.

**Fig. 3:**
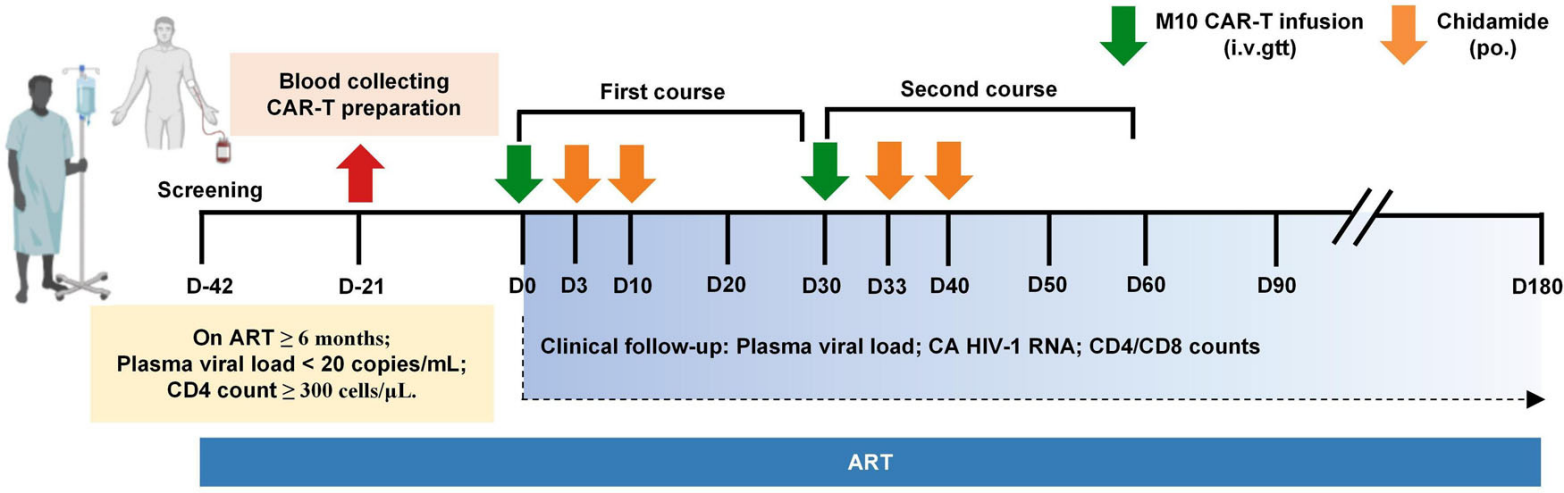
Schematic diagram of the clinical study. The clinical trial included two courses of M10 CAR-T treatment. Each course included a single administration of M10 CAR-T cells and two oral doses of chidamide (30 mg each time) on Day 3 and Day 10 after M10 CAR-T infusions. M10 CAR-T cells were generated from the PBMCs isolated from haplo-identical allogeneic donors. Plasma viral load, CA HIV-1 RNA and CD4/CD8 counts were monitored at regular intervals throughout the study.

Concerning the limited accessibility of the latent viral reservoir by CAR-T cells, we designed a treatment regimen centered on an integration of the ‘shock and kill’ strategy and CAR-T cell infusion. As illustrated in Fig. 3, our regimen consisted of two M10 cell infusions with an interval of 30 days, with each infusion followed by two chidamide stimulations (30 mg per dose). As a histone deacetylase inhibitor (HDACi), chidamide has been recognized as a potent latency-reversing agent (LRA) in the ‘shock and kill’ strategy, with a well-tolerated safety profile^20^. Given that the PBMCs of enrolled participants were not HIV-1 free, we used PBMCs from HLA-haploidentical allogeneic donors, including parents, children, and a sister, to generate M10 CAR-T cells (Supplemental Table S1). We consequently generated M10 CAR-T cells for all enrolled participants, with CAR transduction efficiencies ranging from 10.0% to 81.1% and numbers of infused CAR-positive T cells ranging from 5.0 × 10^7^ to 5.0 × 10^8^, derived according to the dose escalation plan (Supplemental Table S1). Memory phenotypic analysis revealed that stem cell memory T cells (T_SCM_) contributed a high proportion of total infused M10 cells (Supplementary Fig. S3). There was no conditioning regimen before M10 cell infusion for any of the participants.

The study involved a total of 36 person-times of M10 cell infusions, with all the participants showing good tolerance throughout the study. During the trial, we performed electrocardiography assessments for cardiotoxicity and did not observe anomalies such as bradycardia, QT-interval shortening, or ST-segment elevation; meanwhile, all the routine blood and biochemistry test data were within the normal range (Table 2). No infusion toxicity such as fever or decrease in pulse oxygen saturation was observed (Supplementary Fig. S4a, b). No participant exhibited hemodynamic instability with hypotension, tachycardia, or tachypnea (Supplementary Fig. S4c–f). Serum levels of a range of cytokines, including IL-2, IL-4, IL-6, IL-10, TNF-α, IFN-γ, and IL-17A, peaked on Day 31 and were consistently within the normal range (Supplementary Fig. S4g). As analyzed by a serum binding assay for detection of the presence of serum anti-CAR antibody, the majority of participants (11 out of 12) did not show a sign of an ADA response to infused M10 cells, except for Subject 018 (Supplementary Fig. S5). With no indication of treatment-related adverse events (AEs), no participant withdrew from the trial due to a CAR-T-related AE. CD4^+^ T-cell depletion marks one of the primary characteristics of immune homeostasis perturbations associated with chronic HIV-1 infection^21^. The possibility of M10 cell treatment aggravating such a disturbance as a consequence of the clearance of infected CD4^+^ T cells was a concern. Thus, we closely monitored the changes in CD4 counts in all participants during the treatment period. There was no significant difference between CD4 counts at enrollment and those at the final evaluation visit (Supplementary Fig. S6a), in line with the fact that CD4^+^/CD8^+^ T-cell ratios only showed transient alteration following M10 cell infusions and returned to baseline within 7 days (Supplementary Fig. S6b). The persistence and proliferation of M10 cells in peripheral blood were also evaluated. As our results show, M10 cells expanded rapidly after chidamide stimulation. The peak level of modified CAR-T cells detected was 1.92% to 0.33% among PBMCs, and subsequently dropped to < 0.1%. Nevertheless, CAR^+^ T cells were persistently detectable for > 150 days (Supplementary Fig. S7). Together, our data demonstrated that M10 cells have good tolerance and safety profiles in the treatment of PLWH.

**Table 2:** Clinical data of enrolled participants at baseline and the final visit.

	Baseline		Final visit	
	Mean ± SD	Median (Q1 ~ Q3)	Mean ± SD	Median (Q1 ~ Q3)
Hemoglobin (g/L)	147.47 ± 16.03	152.00 (140.00～158.50)	147.47 ± 17.96	152.00 (138.50～162.50)
Red Blood Cell Count (10^12^/L)	4.63 ± 0.47	4.70 (4.12～5.03)	4.71 ± 0.55	4.61 (4.20～5.37)
Platelet Count (10^9^/L)	242.67 ± 60.36	236.00 (204.75～278.00)	240.06 ± 59.81	239.00 (191.50～281.50)
White Blood Cell count (10^9^/L)	7.07 ± 1.74	6.71 (5.36～8.39)	6.42 ± 1.03	6.30 (5.62～7.03)
Absolute Neutrophil Count (10^9^/L)	4.26 ± 1.51	3.72 (3.10～5.37)	3.69 ± 0.94	3.46 (2.98～4.26)
Absolute Lymphocyte Count (10^9^/L)	2.13 ± 0.61	2.11 (1.68～2.62)	2.08 ± 0.42	2.08 (1.80～2.20)
Absolute Monocyte Count (10^9^/L)	0.52 ± 0.13	0.52 (0.41～0.66)	0.48 ± 0.11	0.48 (0.40～0.54)
Absolute Eosinophilia Count (10^9^/L)	0.13 ± 0.11	0.09 (0.07～0.13)	0.13 ± 0.11	0.08 (0.06～0.15)
Absolute Basophil Count (10^9^/L)	0.04 ± 0.01	0.04 (0.03～0.05)	0.04 ± 0.01	0.05 (0.04～0.06)
NA (mmol/L)	139.06 ± 1.47	139.00 (138.00～140.00)	139.00 ± 1.74	139.00 (137.75～140.00)
K (mmol/L)	4.04 ± 0.16	4.00 (3.90～4.19)	4.09 ± 0.22	4.07 (3.95～4.20)
CL (mmol/L)	106.09 ± 1.94	106.90 (104.30～107.15)	106.05 ± 1.92	106.10 (104.55～107.40)
CA (mmol/L)	2.23 ± 0.10	2.23 (2.14～2.31)	2.31 ± 0.13	2.33 (2.23～2.41)
MG (mmol/L)	0.84 ± 0.06	0.82 (0.79～0.89)	0.87 ± 0.06	0.87 (0.84～0.90)
UREA (mmol/L)	4.60 ± 1.33	4.49 (3.63～4.97)	4.75 ± 1.29	4.42 (3.62～6.06)
Creatinine (μmol/L)	72.45 ± 16.27	74.11 (53.87～85.93)	73.49 ± 21.41	69.30 (55.50～83.40)
Total bilirubin (μmol/L)	7.77 ± 4.20	6.30 (4.35～10.60)	10.95 ± 4.88	11.80 (6.20～14.10)
Direct bilirubin (μmol/L)	3.08 ± 1.39	2.80 (2.00～3.55)	3.73 ± 1.53	3.80 (2.00～5.25)
Total Serum Protein(g/L)	75.61 ± 4.07	74.42 (72.52～80.62)	74.60 ± 5.32	73.00 (70.64～78.39)
Serum Albumin (g/L)	45.28 ± 2.17	44.53 (43.76～47.25)	45.76 ± 2.64	44.76 (43.58～48.82)
Alanine-aminotransferase (ALT) (U/L)	29.76 ± 15.31	26.00 (19.00～36.00)	34.15 ± 13.96	37.10 (19.50～45.00)
Aspartate Transaminase (AST) (U/L)	22.00 ± 7.29	19.00 (17.00～25.00)	23.41 ± 5.22	24.00 (19.00～27.05)
Glutamyl Transpeptidase (GGT) (U/L)	53.88 ± 31.54	48.00 (26.00～70.50)	57.41 ± 48.19	47.10 (23.25～72.18)
Lactic Dehydrogenase (LDH) (U/L)	200.59 ± 40.06	188.00 (175.50～228.00)	185.85 ± 24.71	189.00 (171.00～202.50)
Alkaline Phosphatase (ALP) (U/L)	86.06 ± 21.50	87.00 (72.50～102.00)	84.54 ± 19.52	81.00 (70.70～98.50)
β2-microglobulin (μg/L)	1639.58 ± 335.10	1532.28 (1387.67～1798.96)	1631.61 ± 310.24	1519.93 (1373.03～1907.53)
Serum Ferritin (ng/mL)	158.63 ± 149.72	114.62 (60.96～212.68)	88.48 ± 77.30	60.70 (32.75～123.97)

### M10 cell treatment diminished plasma HIV load spiking induced by repetitive administration of chidamide

**Fig. 4:**
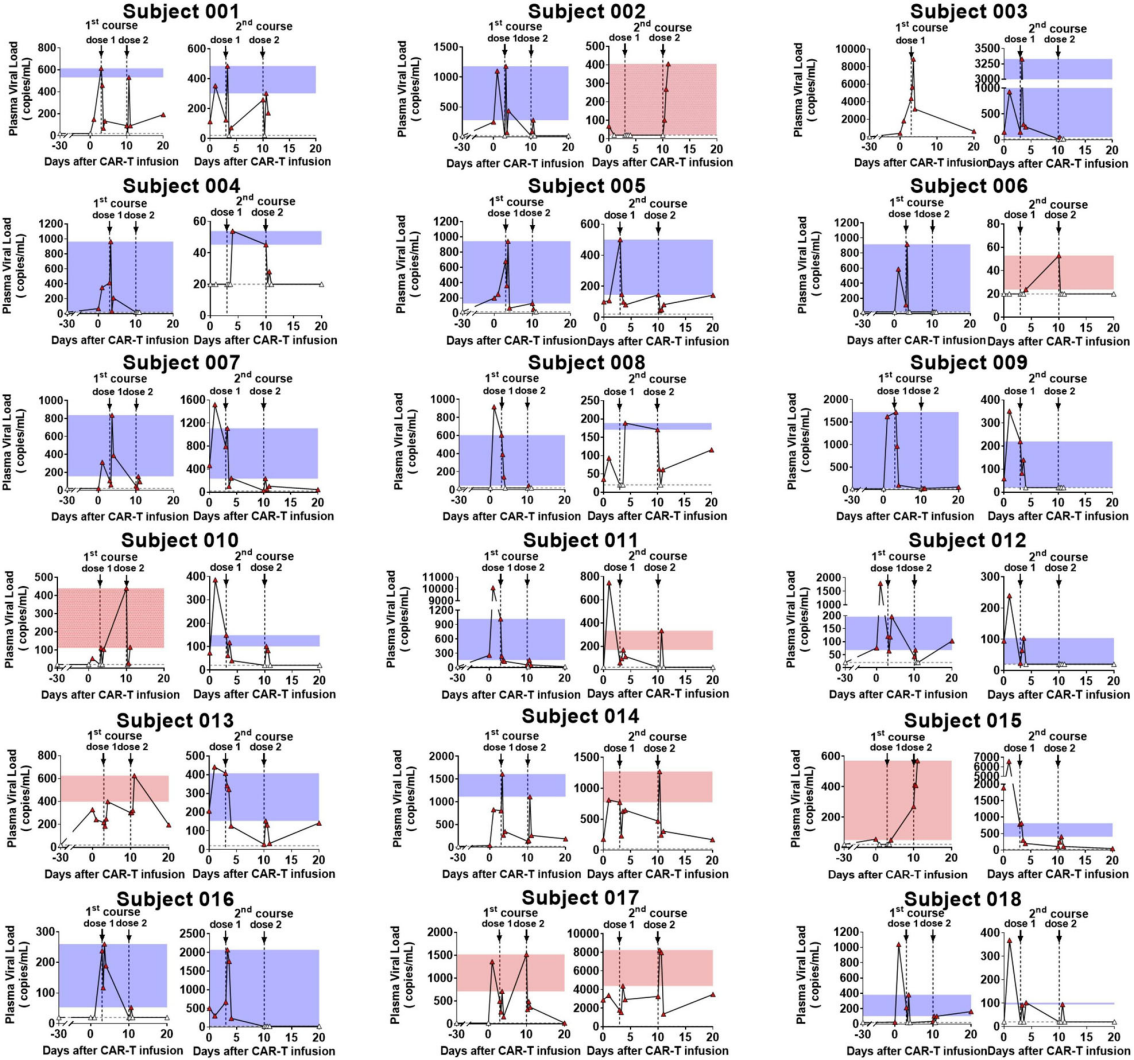
Diminished viral rebound over repeated chidamide administrations after M10 CAR-T treatment. Panels show plasma HIV-1 RNA of study participants after M10 CAR-T infusions. The adoptive transfer of M10 CAR-T cells was performed on Day 0 followed by double administrations of chidamide on Day 3 and Day 10 (shown as black arrows in the panels). All the subjects completed the full two courses of treatment, comprising two doses of chidamide per course, except for Subject 003, who missed the second dose of chidamide during the first course due to personal reasons. Blue shading represents a decline in viral rebound over repeated stimulation, while red shading represents an increase. The limit of detection of HIV-1 RNA levels in this assay is 20 copies/mL (shown as dashed gray lines). Timepoints with an undetectable plasma HIV-1 RNA is indicated by white triangles.

As described above, repeated chidamide administration after M10 cell infusions in our treatment regimen were designed to reactivate the viral reservoir to expose it to M10-mediated clearance. A previous study reported that a single administration of 10 mg of chidamide could reverse virus latency in PLWH undergoing ART, with the induced plasma viremia generally peaking within 24 h^22^. Accordingly, we measured the plasma viral loads at 0, 6, 12, and 24 h after chidamide administration. Subject 003, not taking the second dose of chidamide on Day 10, was excluded from statistical analysis. Our measurements showed that HIV reactivation occurred across the entire study group after the first dose of chidamide, with viremia titers peaking ranging from 48.9 to 1720 copies/mL (Fig. 4). In contrast, the virus reactivation induction effect was significantly subdued in most participants for the second dose of chidamide during the same treatment course. Among the 35 M10 cell infusions, after the second dose of chidamide, 14.3% (5/35) showed no virus reactivation, while 60.0% (21/35) exhibited a lower degree of virus reactivation, with a median decline of 71.1% (range, 7.7% to 98.4%) compared to that after the first dose. Only 25.7% (9/35) of infusions were associated with relatively greater viral reactivation by the second chidamide stimulation than by the first chidamide stimulation.

When examining the dynamics of plasma viral load over the treatment period, we observed that the suppressed virus reactivation by chidamide during the first course generally continued into the second course, with viremia peak values maintained at markedly reduced levels. Among the 18 participants, 15 (83.3%) had a peak viremia level after the final dose of chidamide that was substantially less than that after the first dose, with a median decline of 75.3% (range, 21.1% to 99.4%), while 2 subjects (subjects 010 and 015) showed relatively constant peak viremia values for all 4 doses. A stronger induction of plasma viremia levels by later doses was observed only for 1 subject (subject 017). The most significant clearance of reactivatable HIV was observed in subjects 009, 012, and 016, with the plasma viral load becoming undetectable at the end of the second course.

Overall, two infusions of M10 cells achieved 74.3% treatment efficacy in suppressing the reactivated virus, with a median decline of 62.5%. To determine whether the reduced reactivation of plasma viremia was due to M10 CAR-T cell infusions, we compared our data to a historical control dataset from a previous clinical study with similar enrollment criteria^22^. This historical control dataset included seven participants chronically infected with HIV-1, and the participants received eight oral doses of chidamide without additional immunologic interventions. The data demonstrated sustained chidamide-mediated HIV reactivation throughout the observation period, with peak viremia values essentially the same for the first seven doses in the entire study group and even higher after the eighth dose in six out of the seven participants^22^. Thus, we conclude that the significantly suppressed viral titer spiking in participants in response to M10 cell infusions reflects the high potency of M10 cells in clearing replication-competent viruses once reactivated from latency by chidamide.

### M10 cell treatment led to a significant reduction in the size of the viral reservoir

**Fig. 5:**
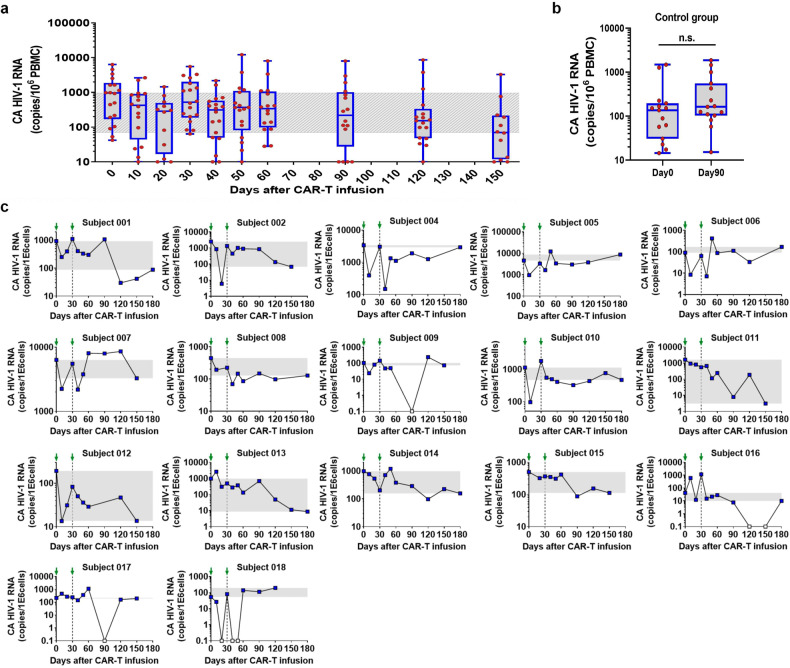
The viral reservoir was significantly decreased in HIV-1 infected subjects after adoptive transfer of M10 CAR-T cells. **a** Panel shows a summary of the changes in CA HIV-1 RNA levels after M10 treatment at indicated time points (*n* = 18). **b** Panel shows changes in CA HIV-1 RNA levels in people who only received conventional antiviral therapy at indicated time points (*n* = 15). **c** CA HIV-1 RNA levels (copies per million PBMCs) of each participant after M10 CAR-T infusions. The adoptive transfer of M10 CAR-T cells was performed on Day 0 and Day 30 (shown as green arrows in the panels). Timepoints with undetectable CA HIV-1 RNA levels are indicated by white squares. Gray shading represents the change in CA HIV-1 RNA from baseline.

To further evaluate the efficacy of M10 cell infusions, we next sought to track the changes in viral reservoir size over the course of the clinical trial. There has been substantial evidence supporting cell-associated HIV-1 RNA (CA HIV-1 RNA) as a reliable proxy for viral reservoir size^23,24^. Therefore, we measured the levels of CA HIV-1 RNA in PBMCs in all participants before and after M10 cell treatment. The median baseline levels of CA HIV-1 RNA, expressed as copies per million PBMCs, was 955 (range, 42 to 6330) (Fig. 5a). During the first course of M10 CAR-T cell treatment, this value fell to 288 on Day 20 (*P* = 0.0034) but rebounded in 11 out of 18 subjects on Day 30. After the second M10 cell infusion, a rapid decline in CA HIV-1 RNA occurred again, with the median value decreasing from 522 on Day 30 to 311 on Day 40 (*P* = 0.0071). When these results were correlated with the plasma viral loads, we found that successful latency reversal might be required to achieve a notable reduction in CA HIV-1 RNA levels. For instance, subject 015 showed limited viral load spiking in response to all four doses of chidamide, coincident with no substantial alteration in CA HIV-1 RNA levels during both courses of M10 cell treatment.

Among the 18 enrolled participants, 17 completed a follow-up that was terminated on Day 150. Analyses of the dynamic changes in CA HIV-1 RNA levels during this extended period resulted in the division of the study cohort into two groups. The first group included 10 subjects (58.8% of the cohort) who shared a persistent and stable reduction in CA HIV-1 RNA levels. The average order of magnitude of reduction at the final visit relative to baseline was 1.15 (range, 0.38 to 2.74), with 2 (subjects 011 and 013) and 3 (subjects 001, 002, and 012) subjects showing an over 100-fold and over 10-fold decrease in viral reservoir size, respectively (Fig. 5c). The second group included 7 participants (41.2% of the cohort) characterized by a CA HIV-1 RNA level at the final visit similar to that at baseline. As reported previously, spontaneous changes above 2.11-fold in CA HIV-1 RNA levels occurred in only < 5% of repeated measurements among individuals undergoing long-term ART^25^. Our results also showed that people who only received conventional antiviral therapy demonstrated stable CA HIV-1 RNA levels within 90 days (Fig. 5b). Additionally, in the aforementioned historical control study, all 7 participants completing the eight oral doses of chidamide demonstrated no significant change in CA HIV-1 RNA levels^22^. Hence, the reductions in CA HIV-1 RNA levels observed in our study subjects were most likely ascribable to the adoptive transfer of M10 cells. These analyses further supported our hypothesis that M10 CAR-T cell infusion followed by repeated chidamide administration was effective in shrinking the latent viral reservoir in PLWH.

### M10 CAR-T cell infusion exerted selective pressure on the HIV reservoir, as revealed by third-generation sequencing

**Fig. 6:**
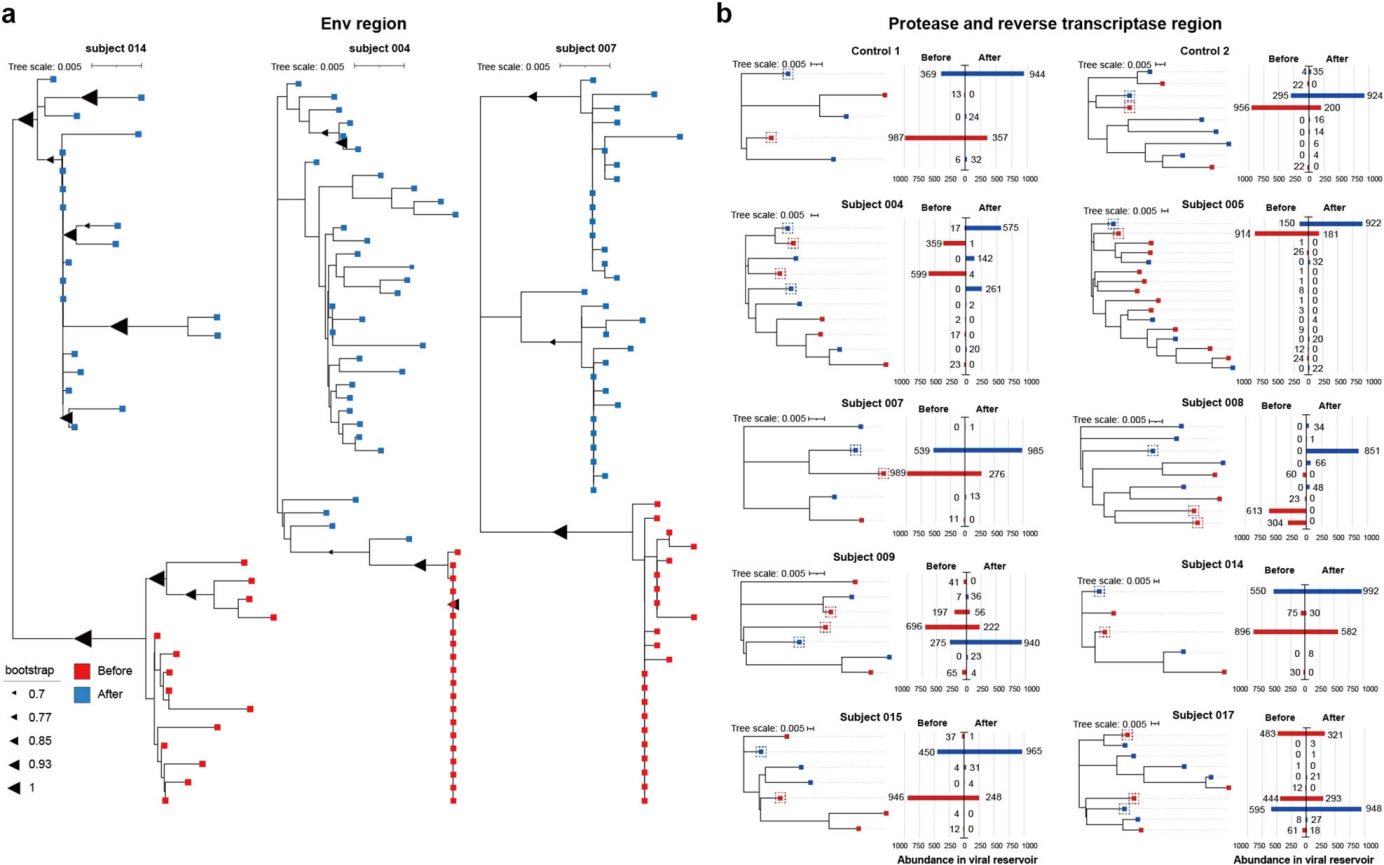
Selective pressure mediated by M10 CAR-T cells. **a** Neighbor-joining phylogenetic trees were constructed using aligned *Env* sequences amplified from replication-competent viruses in latent reservoir prior to (red) or post (blue) M10 CAR-T treatment. **b** Maximum likelihood phylogenetic trees were constructed using aligned protease and adverse transcriptase (PR/RT) region sequences. Bar graphs show the abundance of each identical sequence and the population it represents in pre-treatment or post-treatment viral reservoir. Sequences from pre-treatment samples are shown in red, and sequences from post-treatment samples were shown in blue.

Having confirmed the efficacy of M10 cell treatment in reducing the size of the HIV reservoir, we next sought to determine the action of M10 cells in restricting the HIV population at the genetic level. To this end, we first analyzed the *env* sequences present in replication-competent viral populations from the latent reservoir at baseline or the final visit. Among 18 study subjects, we successfully obtained replication-competent viruses from only three subjects at both sampling time points, and the corresponding specimens were subjected to RNA extraction and then amplification of *env* genes by RT‒PCR. The obtained PCR product was then subjected to PacBio sequencing, followed by analysis of the evolutionary relationships of the derived sequencing reads that passed quality control. A phylogenetic tree analysis revealed that the *env* sequences from the posttreatment sample were significantly distinct from the pretreatment sequences, segregating into different subclades. Intriguingly, the posttreatment populations appeared to display a mean branch length shorter than the pretreatment populations, indicative of an ancestral state relative to the latter on the evolutionary trajectory (Fig. 6a). These results were consistent with a scenario in which M10 CAR-T cells effectively eliminated the dominant latent HIV species and thus the corresponding *env* genes after induction by chidamide, consequently allowing the evolutionarily older viral species, which existed as an underrepresented subpopulation, to repopulate, particularly after waning of infused M10 cell levels.

We also examined the effect of M10 CAR-T cell treatment on HIV-1 internal genes by applying the same third-generation sequencing to the *pol* gene amplified from patient PBMCs at baseline or the final visit. The constructed phylogenetic trees showed a shift in the composition of the viral reservoir after M10 cell treatment for most participants. The dominant *pol* genes in the pretreatment viral pool shrank or even disappeared after M10 cell treatment, concomitant with expansion of previously nondominant versions, a pattern similar to that of *env* genes derived from replication-competent HIVs as described above (Fig. 6b). In contrast, the control subjects receiving only ART demonstrated no significant change in the genetic diversity of the *pol* gene after antiretroviral therapy. These data further reinforce the hypothesis that M10 CAR-T cells exerted profound pressure on the *env* gene, to some extent mirrored in the coupled HIV-1 internal genes, resulting in selective purging of the replication-competent HIV reservoir in PLWH.

## Discussion

In this study, we presented the development of a novel type of multifunctional anti-HIV-1 CAR-T cell, namely, M10 cells, and the preliminary results regarding their anti-HIV-1 efficacy in PLWH obtained from a phase I clinical trial. The multifunctional nature of M10 cells renders them more capable than monofunctional CAR-T cells in overcoming challenges associated with using CAR-T-cell therapy to treat HIV-1 infection. Although it awaits future investigations of whether the two ancillary functionalities of M10 cells, bNAb-mediated neutralization of free HIV-1 to protect uninfected cells, including infused CAR-T cells, and CXCR5-mediated germinal center targeting, operate in vivo in M10-treated patients, our in vitro characterizations firmly corroborated their utilities.

The greatest challenge in HIV infection treatment lies in the limited accessibility of the latent virus reservoir created by the integration of the reverse-transcribed viral genome into host DNA in the form of a provirus. This reservoir has multiple sources, including not only the “T-cell” reservoir existing among memory CD4^+^ T cells but also “myeloid-cell” and “nonimmune-cell” reservoirs associated with monocytes/macrophages/dendritic cells and astrocyte/microglia/epithelial cells, respectively^26^. This inaccessibility of HIV-1 promoted the development of the “shock and kill” strategy, which is intended to force latent virus out of its sanctuary and accessible to subsequent eradication^27–30^. In our clinical trial, we adopted a two-course regimen inspired by the “shock and kill” strategy, with one treatment course comprising a single M10 cell infusion followed by two administrations of the latency-reversing agent (LRA) chidamide. Our results showed that this regimen achieved 74.3% efficacy in suppressing chidamide-induced plasma viral titer spikes, concomitant with a mean reduction of 48.6% in CA HIV-1 RNA levels. The reactivation capacity of LRAs showed a correlation with the size of the HIV-1 reservoir^31^. In this respect, the ability of M10 cells to efficiently eliminate the reactivated viral reservoir aligns well with the observed potential differences in the magnitude of induced viremia between the two doses of chidamide treatment, which are likely a reflection of the efficient M10-mediated purge of the HIV-1 reservoir reactivated by the action of the first dose of chidamide. As a comparison, the previous REDUC trial reported an average 39.7% reduction in total HIV-1 DNA levels but no significant change in CA HIV-1 RNA levels^32,33^. Another clinical trial (NCT02616874) used a combinatorial treatment of the LRA romidepsin and MVA.HIVconsv vaccine following the “shock and kill” strategy, reporting only a modest reduction in the viral reservoir size^34^. The success of our regimen demonstrated the potency of M10 cells in clearing HIV-infected cells once these cells express the targeted Env protein on the surface. Indeed, M10 cells appeared to have superior anti-HIV activities compared with previously reported bNAb-derived CAR-T cells, as evidenced by their ability to more robustly reduce the size of the viral reservoir, especially in patients with lower baseline CA HIV-1 RNA levels^8^.

Notably, chidamide, similar to other LRAs currently used or under investigation, mainly reactivates the latent T-cell reservoir. Its ability to reverse viral latency in the myeloid cell or non-immune cell reservoir in vivo is largely unclear^20,22,35^. Thus, in our regimen, the action of M10 cells on HIV-1 reservoirs might be restrained to the T-cell compartment, as ineffective latency reversal could hinder their access to myeloid-cell and/or non-immune-cell reservoirs. On the other hand, reservoir cells with defective proviruses that do not express Env or express Env lacking the M31 CAR-targeting region might also resist M10 attack even if reactivated. These remaining proviruses persistently produce CA HIV-1 RNA and may expand as a consequence of antigen-driven clonal proliferation or homeostatic proliferation of infected cells^36–38^. Thus, the properties of M10 cells as Env-specific targeting CAR-T cells could be a factor underlying the discrepancy that we observed between a significant reduction in the plasma viral titer rebound and reduced containment of CA HIV-1 RNA. Genetic evidence, albeit from a limited number of samples, further corroborated this explanation by showing a more profound pressure by M10 cells on *env* sequences of replication-competent viruses derived from the T-cell reservoir than on HIV internal genes associated with PBMCs. We therefore propose that stemming from the regimen presented in this study, future exploration of synergistic or sequential shock using a combination of mechanistically distinct LRAs may further augment the treatment efficacy of M10 cells. Moreover, further studies should be done to show if the selected viruses have become relative resistant to M10 in the future. On the other hand, it is also worth noting that HIV-1 RNA levels were only indirect suggestion of HIV-1 reservoir size which could due to altered HIV-1 LTR activity or number of HIV-1-infected cells. Thus, more efforts should also be made to directly assess the effect of M10 cells on HIV-1 reservoir, which could benefit from developing standard assays capable of differentiating and quantifying free and cell-bound HIV-1 viruses in the presence of delivered lentiviral-based CAR-T cells. Additional improvement may come from coupling M10 cells with anti-HIV-1 TCR-T cells to expand the recognition and killing of HIV-infected cells with intracellular viral antigen expression.

In summary, we presented both in vitro and preliminary clinical data supporting the potential of M10 cells as a novel cellular therapy against HIV infection. Based on our demonstration of M10 cell efficacy for allogenic CAR-T cells through a four-dose regimen, there is a reason for optimism that a need-based repetitive administration of M10 cells alongside LRAs can be developed into a new therapeutic option toward a functional HIV cure.

## Materials and methods

### Cell lines

The following cell lines were used: MT4, A549, TZM-bl, ACH2. All cell lines were purchased from ATCC and cultured in RPMI 1640 (Corning #10-040-CVR) supplemented with 10% fetal bovine serum (FBS) (BI #04-001-1acs) and 1% penicillin-streptomycin (Corning #30-002-CI), except for A549 cells and TZM-bl cells, which were cultured in DMEM (Corning #10-013-CV) supplemented with 10% FBS and 1% penicillin-streptomycin. Lentiviruses carrying the HIV-1 gp145 gene were delivered into MT4 and A549 cells to generate MT4-gp145 and A549-gp145, respectively. All the cells were maintained in a humidified atmosphere containing 5% CO_2_ at 37 °C.

### CAR construction

DNA encoding the fusion protein consisting of human CD8 signal peptide (NP_001759.3 aa 1–21), Flag tag (DYKDDDDK), m36.4 peptide, 3× G_4_S linker, mD1.22 peptide, the hinge spacer, transmembrane and signaling domain of human CD28 (NP_006130.1 aa 114–220), human 4-1BB signaling domain (AAA53133.1 aa 209–255), and human CD3ζ signaling domain (NP_932170 aa 52–164) in N- to C-terminal order was cloned into the lentiviral transfer plasmid (ABpCCLsin_EF1_MCS_P2A_EGFP) to generate M31 CAR (ABpCCLsin_M31-P2A-EGFP). Replacing the EGFP encoding sequence with the expression cassette encoding the 10E8scFv-Fc antibody in M31 CAR generated the ABpCCLsin_M31-P2A-10E8scFv-Fc vector, which was used as the CAR in M10 cells. The CXCR5-expressing lentiviral vector, ABpCCLsin_CXCR5, was derived from ABpCCLsin_EF1_MCS by inserting DNA encoding CXCR5 (NP_001707.1 aa 1-372) into the multiple cloning site. The protein sequences for the composite parts of CAR construct were provided in Supplemental Table S2.

### Generation of CAR-T cells

Allogeneic PBMCs were isolated from 100 mL peripheral blood of donors and stored in liquid nitrogen. After thawing, PBMCs were cultured in T cell growth medium (TGM), which is derived from X-VIVO 15 culture medium (Lonza #BE02-060F) by addition of human IL-7 (R&D systems #P13232), human IL-15 (R&D systems #P40933), and human IL-21 (Novoprotein #GMP-CC45). For lentiviral transduction, PBMCs were first stimulated for 48–72 h with anti-hCD3 and anti-hCD28 coated immunobeads at a bead-to-cell ratio of 1:1, followed by infection with concentrated lentivirus (produced by Hangzhou Kanglin Biotechnology Company) via co-centrifugation method (1000× *g* for 90 min). The culture medium was changed to fresh TGM the next day and the immunobeads were removed 4–5 days post-transduction. The cell density was adjusted to 0.5–2 × 10^6^ /mL every 2–3 days until the required cell dose was reached.

### Flow cytometry

M10 CAR-T cells (5 × 10^5^) were harvested, washed, and stained with PE-conjugated anti-DYKDDDDK (Biolegend #637310) and AF488-conjugated Hu CXCR5 antibody (BD Biosciences #558112) at 4 °C for 30 min. Data were acquired with a BD LSRFortessa flow cytometer (BD Biosciences) and analyzed using FlowJo software.

### In vitro assessment of cytokine production capability of CAR-T cells

The indicated CAR-T cells (1 × 10^5^) were co-cultured with gp145 expressing or non-expressing MT4 cells at an effector-to-target ratio of 1:1 in a 96-well round-bottom plate. After 24 h, the co-culture supernatant was collected and stored at –20 °C for later quantitation of IFN-γ and IL-2 levels using a human IFN-γ ELISA Set (BD Bioscience #555142) and a human IL-2 ELISA Set (BD Bioscience #555190).

### In vitro assessment of CAR-T cell cytotoxicity

A549 cells expressing with or without gp145 protein were plated in duplicate in E-Plate 16 PET (Agilent #20201031) at a density of 1 × 10^4^ cells/well and grown for 14–16 h. CAR-T or UTD cells were then added to the plate at an E:T ratio of 1:1.

For TZM-bl assay, the TZM-bl cells were seeded at 1 × 10^4^ cells/well and infected with HIV-1 pseudoviruses at 200 TCID50/well for 24 h. After removing the supernatants, CAR-T or UTD cells were added to the plate at an E:T ratio of 1:1, with target cells only serving as the control. The dynamic changes in cell index (CI) values were recorded using the XCelligence RTCA system. The formula used to calculate the percent normalized cytotoxicity is % Lysis = (control group CI − experimental group CI)/control group CI × 100.

PMA-activated ACH2 cells were labeled with *eflour450* and cocultured with UTD or CAR-T cells at an E:T ratio of 1: 1. The percentage of *eflour450*-labeled target cells was measured at 0 h and 24 h through flow cytometry. The formula used to calculate the percent normalized cytotoxicity is as follows: % Lysis = (target cells % at 0 h − target cells % at 24 h)/ target cells % at 0 h × 100.

### Detection of p24 antigen

The supernatant concentrations of the p24 antigen were assessed by ELISA using an HIV-1 p24 Antigen Detection Kit purchased from the Biomedical Engineering Center of Hebei Medical University.

### In vitro cell migration assay

The assay was performed with a 24-well transwell culture plate (5 μm pore size, Corning) by adding control or transduced T cells (1 × 10^5^ cells/well) to the upper chamber and 600 μL serum-free medium supplemented with different concentrations of CXCL13 to the lower chamber. With the plate placed in a humidified 37 °C, 5% CO_2_ incubator, the densities of cells migrating into the lower chamber were measured at 2 h, 4 h, and 6 h time points to calculate migration rates.

### Study design and participant characteristic

The objective of our study was to estimate the safety and efficacy of treatment with M10 CAR-T cell infusions in PLWH undergoing ART. We generated allogenic M10 CAR-T cells from haploidentical, including mainly parents and children, except for one case, where a sister was the donor. High-resolution human leukocyte antigen (HLA) typing, covering HLA-A, HLA-B, HLA-Cw, HLA-DRB1, and HLA-DQB1 loci, were performed in cases where donors were unrelated to their recipients to confirm that they are haplo-identical. As for participants with related donors, including parents and children, the HLA typing step was omitted. The resultant M10 CAR-T cells, after verification of transducing efficacy, were transferred to recipients, who were PLWH receiving successful ART treatment, as characterized by clinically stable on ART regimen for at least 6 months with undetectable HIV-1 RNA level and a CD4 count ≥ 300 cells/μL at enrollment. Study participants have not undergone any additional immunotherapeutic intervention besides ART. The study used a standard 3 + 3 design to establish the maximum tolerated dose: an initial cohort received either 1 × 10^6^ CAR-positive T cells per kg (low dose), 1 × 10^7^ CAR-positive T cells per kg (high dose), or the entire CAR-T product in case of the total numbers of generated CAR-T cells do not reach the assigned dose; the maximum body weight for dose calculation was set at 50 kg. Following the dose-escalation phase, an expansion cohort was treated at the maximum tolerated dose.

### Determination of cell-associated and plasma HIV-1 RNA

For the determination of cell-associated HIV-1 RNA, total RNA was extracted from peripheral blood mononuclear cells (PBMCs) using a Magen HiPure Total RNA Plus Mini Kit (Guangzhou, China) according to the manufacturer protocol, followed by the quantification of the contained HIV-1 RNA using a SUPBIO HIV-1 usRNA Quantitative PCR kit (Guangzhou, China). Plasma HIV-1 RNA was determined using the Cobas AmpliPrep/Cobas TaqMan HIV-1 Test (version 2.0) following standard procedures. A third-party laboratory performed the two determinations.

### Viral outgrowth assay

Amplification of replication-competent viruses was performed according to a previously described protocol^39^. In brief, 1 × 10^6^ CD4^+^ T cells were isolated from patient PBMC samples and then co-cultured with 1 × 10^7^ irradiated PBMCs derived from healthy donors in RPMI160 media supplemented with 10 ng/mL IL-2 and 1 μg/mL PHA. On Day 2, PHA was removed, followed by adding CD4^+^ T cells from uninfected donors, namely the first CD4^+^ blasts, as target cells for viral outgrowth. The second CD4^+^ blasts were added on Day 9. Cultures were collected on Day 14 and tested for the presence of HIV-1 using an HIV-1 p24 antigen detection kit.

### Polymerase chain reaction (PCR) amplification of *pol* and *env* gene

PCRs were performed according to previously described protocols^40,41^. The template for the *pol* gene (*protease/ reverse transcriptase* (PR/RT) region) amplification was total DNA extracted from patient PBMCs, and that for the *env* gene was the reverse transcription product of RNA of CD4^+^ T cell-derived replication-competent virus acquired through VOA. The PR/RT region (HXB2 nucleotides 2242 to 3312) was amplified by two-round nested PCR, using primer pair of 5′-CAAGGGAAGGCCAGGGAATTT-3′ and 5′-TGACCC-ATCAAAAGACTTAATAGCAGAAATA-3′ in the first round, and 5′-CCT-TTAACTTCCCTCAGGTCACTCT-3′ and 5′-ATTGACAGTCCAGCTGTC-TTTTTCTG-3′ primer pair in the second round. The amplification of the *env* gene (HXB2 nucleotides 6225 to 8795) was also achieved via nested PCR by using 5′-TAAGCCTCAATAAAGCTTGCCT-TGAGTGC-3′ and 5′-CTGCTAATCAGGGA-AGTAGCCTTGTGT-3′ primer pair in the first round, and 5′-AGCCTTAGGCATC-TCCTATGGCAGGAAGAAG-3′ and 5′-GTCTCGAGATGCTGCTCCCACCC-3′ primer pair in the second round. All the PCR reactions were performed using PRIMESTAR MAX DNA polymerase (TAKARA). The cycling conditions for the first round of nested PCR were: 98 °C for 3 min; 35 cycles of 98 °C for 10 s, 55 °C for 30 s, and 72 °C for 30 s; 72 °C for 7 min. The second round of nested PCR used 1 μL of the PCR product from the first round as the template with the cycling conditions set at: 98 °C for 3 min; 45 cycles of 98 °C for 10 s, 55 °C for 30 s, and 72 °C for 20 s; 72 °C for 7 min. An aliquot of the final PCR product was analyzed by electrophoresis on a 1% agarose gel, stained with ethidium bromide, and visualized under ultraviolet light to confirm the expected amplicon size.

### PacBio sequencing

The PCR products of HIV genes were sent to Genewiz (Genewiz, Suzhou, China) for PacBio library construction and sequencing. For each sample, > 2 μg purified PCR fragments were used for library preparation. In brief, fragmented DNAs were treated with End Prep Enzyme Mix that simultaneously catalyzes end repairing, 5′ phosphorylation, and 3′ dA-tailing, followed by adding universal hairpin adapters to both ends using a Blunt/TA Ligase. The resultant SMRTbell libraries were quantified by a Qubit 3.0 Fluorometer and profiled by an Agilent 2100 Bioanalyzer (Agilent Technologies, Palo Alto, CA, USA) before sequencing on a *PacBio* sequel instrument according to the manufacturer’s instruction (Pacific Biosciences of Califonia, Inc., California, USA). The sequencing data and the derived protein sequences have been submitted to the NCBI Bioproject under accession number PRJNA903552.

### PacBio sequencing data processing and evolutionary analysis

The raw PacBio subreads were subjected to Circular Consensus Calling to generate high-quality circular consensus (CCS) reads using *pbccs* software (version 3.3.0). After characterization through statistical analyses to obtain information such as read numbers, average read length, and GC content, the CCS reads were split guided by the flanking primer pair using *lima* software (version 1.9.0), and subsequently used for evolutionary analysis. For phylogenetic tree construction, after the removal of those with premature stop codons or frameshift mutation in the protein-coding region, the sequences were analyzed by the CD-HIT program for hierarchy clustering with a clustering threshold of 95%, where the longest amino acid sequence within each cluster was selected as the representative sequence for comparison^42^. Phylogenetic trees were constructed using MEGA v11 with a bootstrap test of 1000 replicates based on the Maximum Likelihood method^43^. Interactive Tree of Life software (iTOL v6) was used to visualize the phylogenetic tree and draw bar graphs depicting sequence abundance^44^. The processed data and the involved in-house scripts were deposited in *figshare* platform (https://figshare.com/articles/online_resource/figshare_M10_CAR-T/21671042) and *Github* (https://github.com/Ryan-zhu-sudo/github_M10-CAR-T), respectively.

### Serum binding assay

The method used to detect anti-CAR antibodies was performed according to a previously described protocol^45^. Briefly, the serum was heat inactivated for 30–35 min at 56 °C. Cryopreserved transduced CAR-T cells, as well as mock transduced PBMC, were thawed and used directly as target cells. Serum was added to wells containing 1 × 10^5^ CAR or mock transduced T cells for a final dilution of 1:10. The serum and cells were incubated at 4 °C for 20 min and then washed twice to remove unbound antibodies of the serum. To measure bound IgG, cells were stained with live/dead, anti-human CD3, anti-Flag tag and anti-human IgG. Following the 20-min antibody staining, cells were fixed with 1% paraformaldehyde and detected by flow cytometry.

### Statistical analysis

All statistical analyses were performed using GraphPad Prism 7.0 (GraphPad Prism Software Inc., San Diego, California). The data were presented as the mean ± SEM unless otherwise described. Statistical differences were determined by paired Student’s *t*-test (two-tailed) and one-way ANOVA with Tukey’s test when analyzing the significance of differences among three or more groups. With a *P*-value of <0.05 considered statistically significant, statistical significances were reported as *P < 0.05, **P < 0.01, ***P < 0.001, or ****P < 0.0001.

### Study approval

Our clinical trial was approved by the Ethics Committee of Shanghai Public Health Clinical Center and registered at chictr.org.cn (ChiCTR2000028826). Written informed consent was presented before enrollment and signed by all the patients participating in the clinical trial. The study was conducted in accordance with legal and regulatory requirements and the general principles outlined in the International Ethical Guidelines for Biomedical Research Involving Human Patients, Guidelines for Good Clinical Practice, and the Declaration of Helsinki.

### Supplementary information

Supplementary Information
