# Supplementary material for: Efficacy and safety of novel multifunctional M10 CAR-T cells in HIV-1-infected patients: a phase I, multicenter, single-arm, open-label study
Source: Cell Discov. 2024 May 14;10:49. doi: 10.1038/s41421-024-00658-z (PMC11091177; doi:10.1038/s41421-024-00658-z)
Supplement: Supplementary file 1 — Supplementary Information [file 41421_2024_658_MOESM1_ESM.pdf]

Supplementary Fig. S1

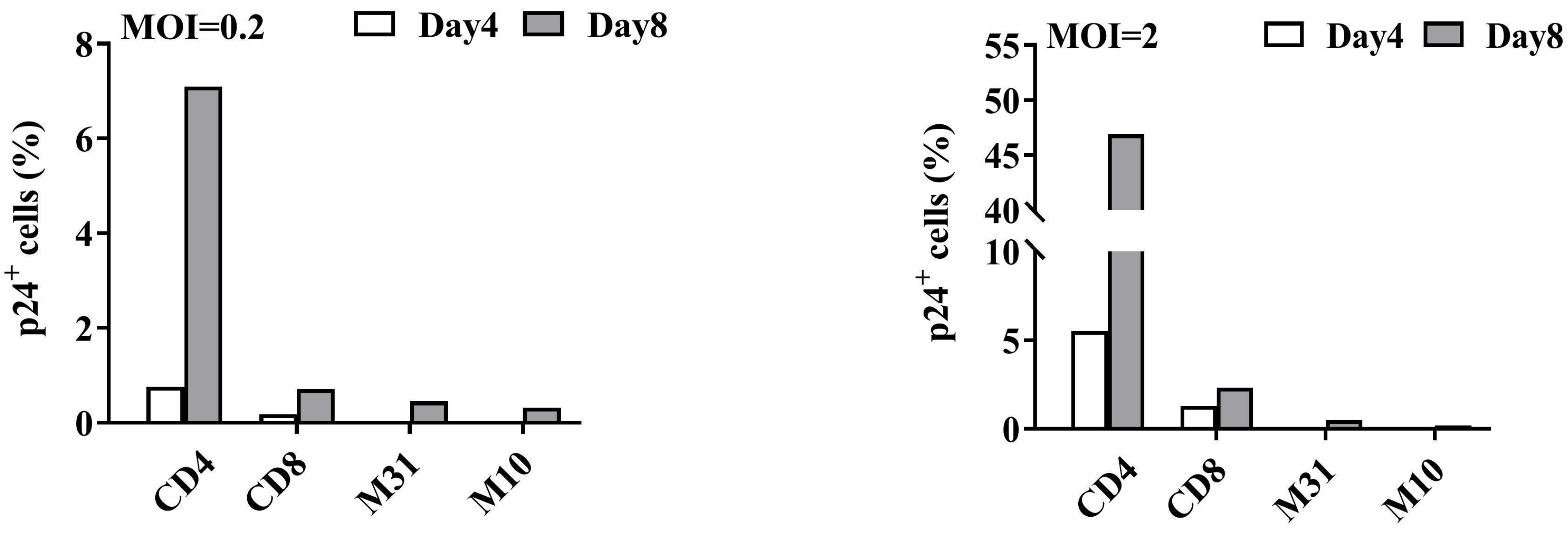

Supplementary Fig. S2

a

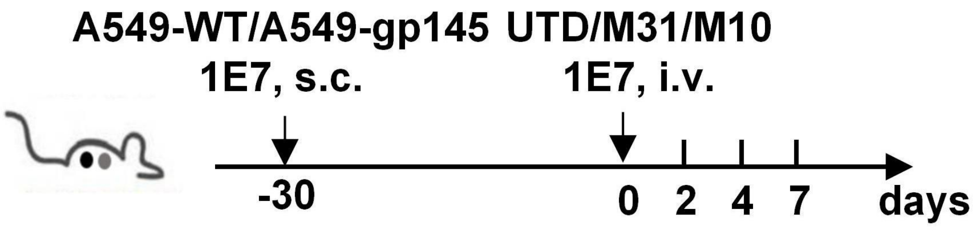

b

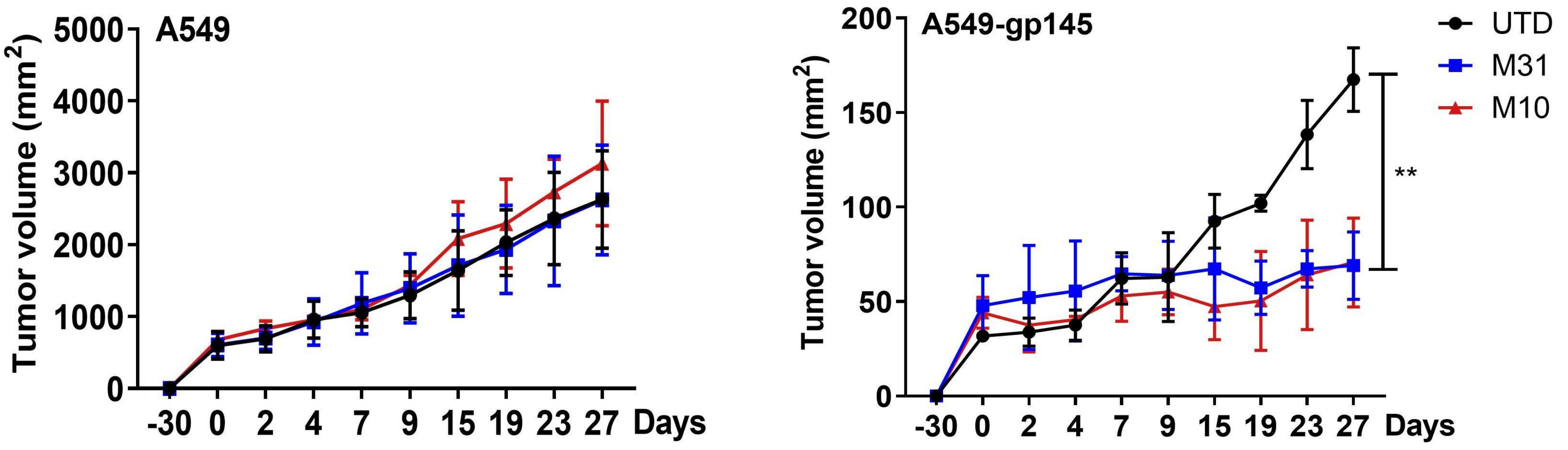

Supplementary Fig. S3

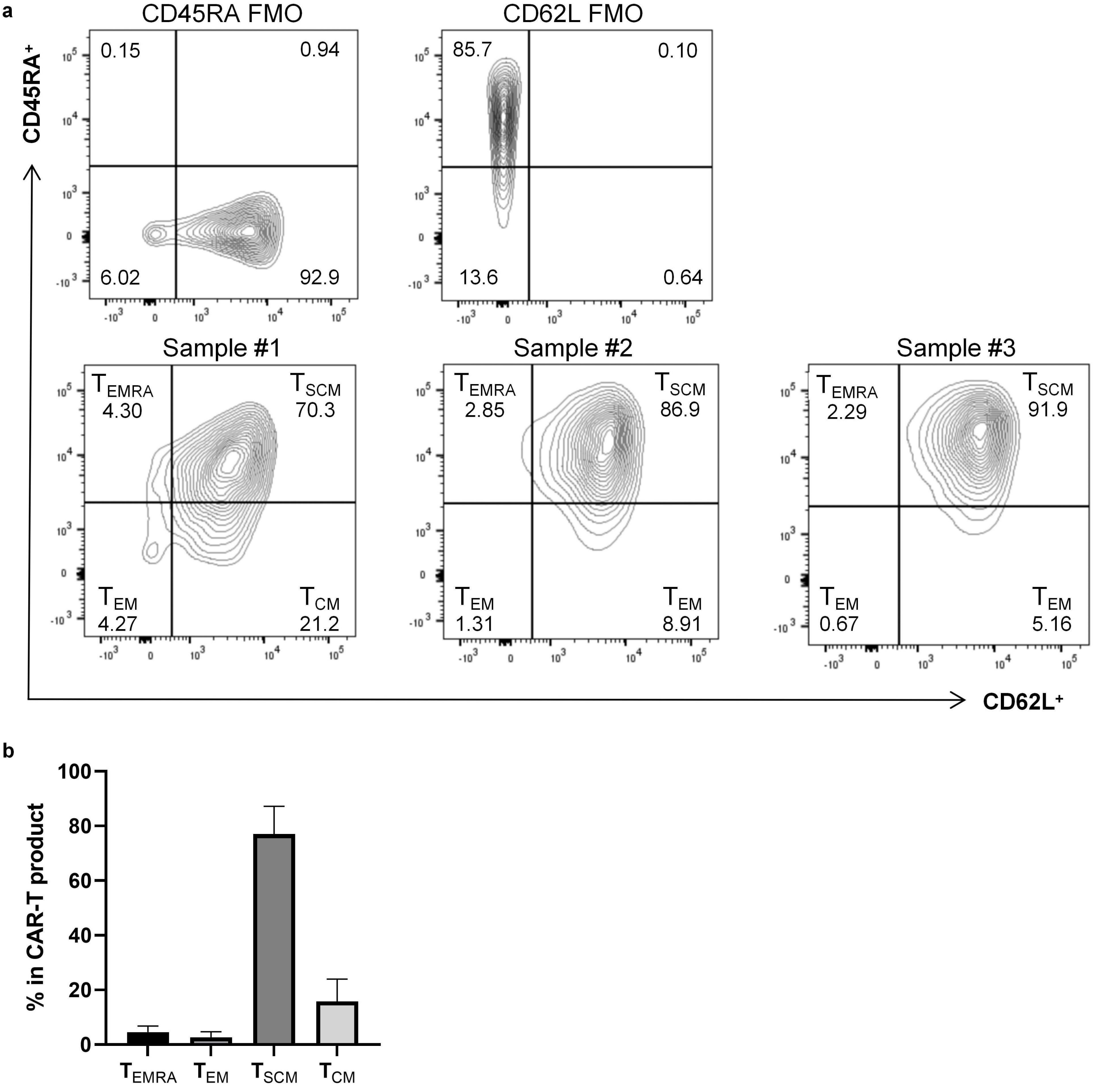

Supplementary Fig. S4

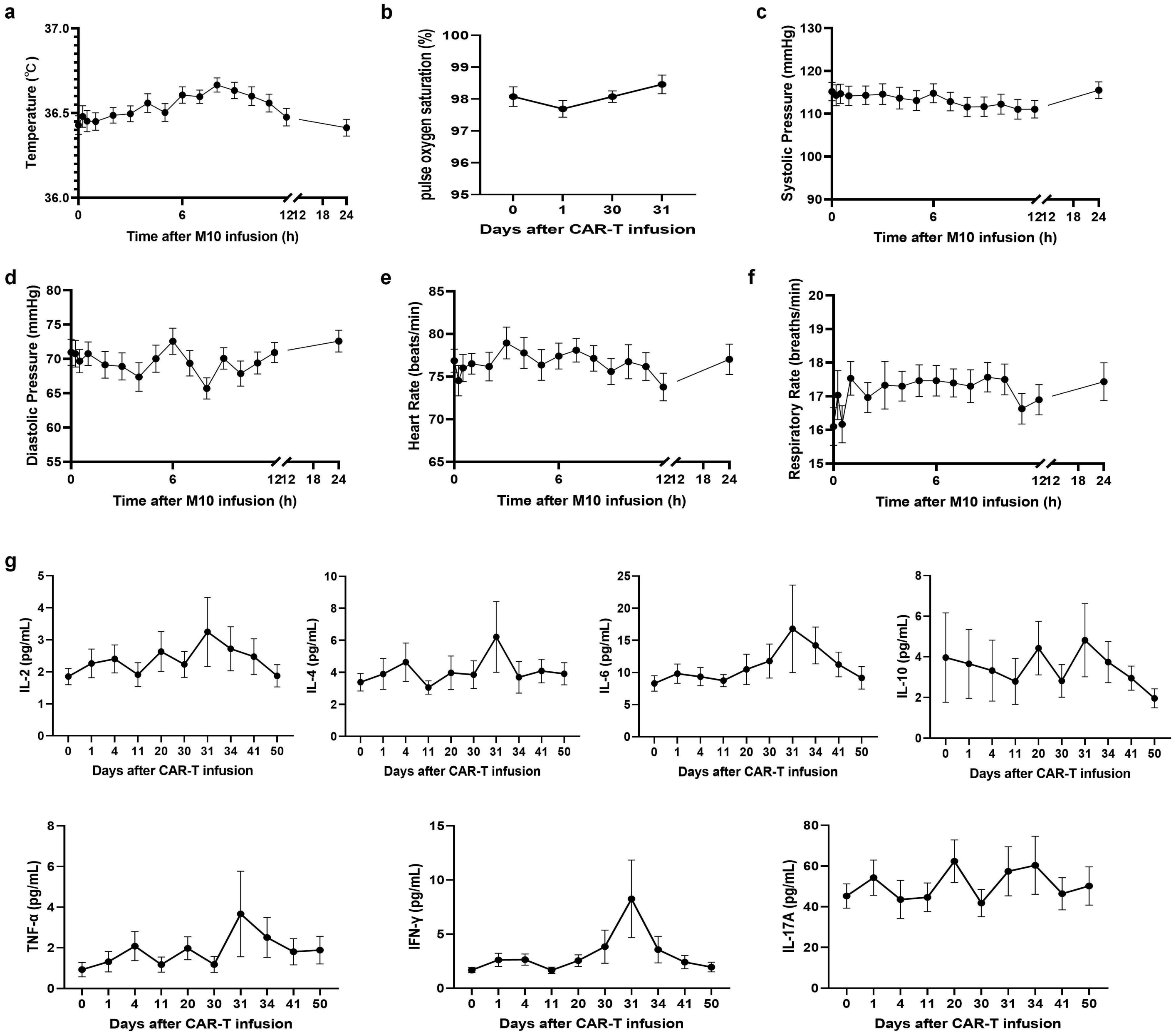

Supplementary Fig. S5

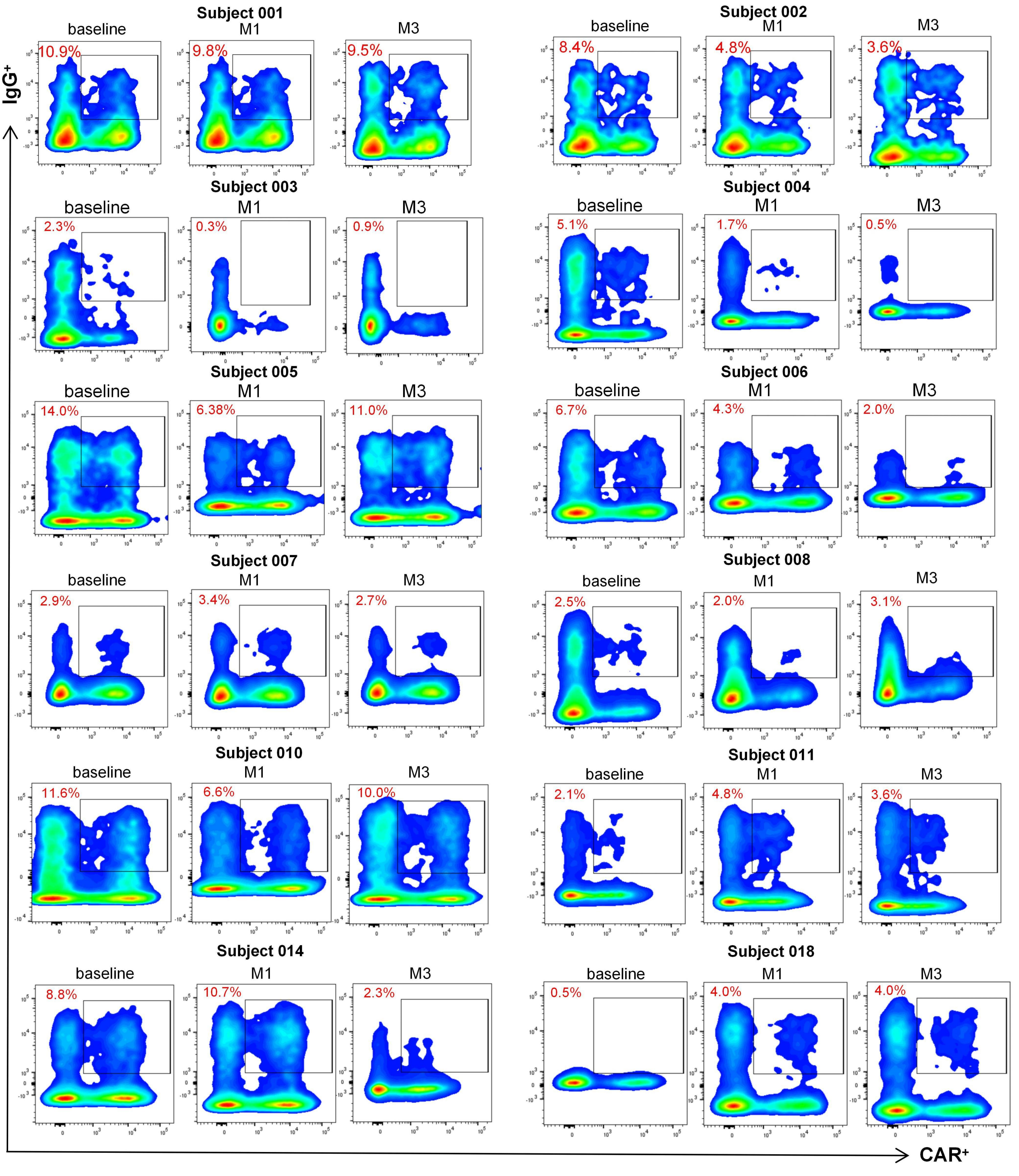

Supplementary Fig. S6

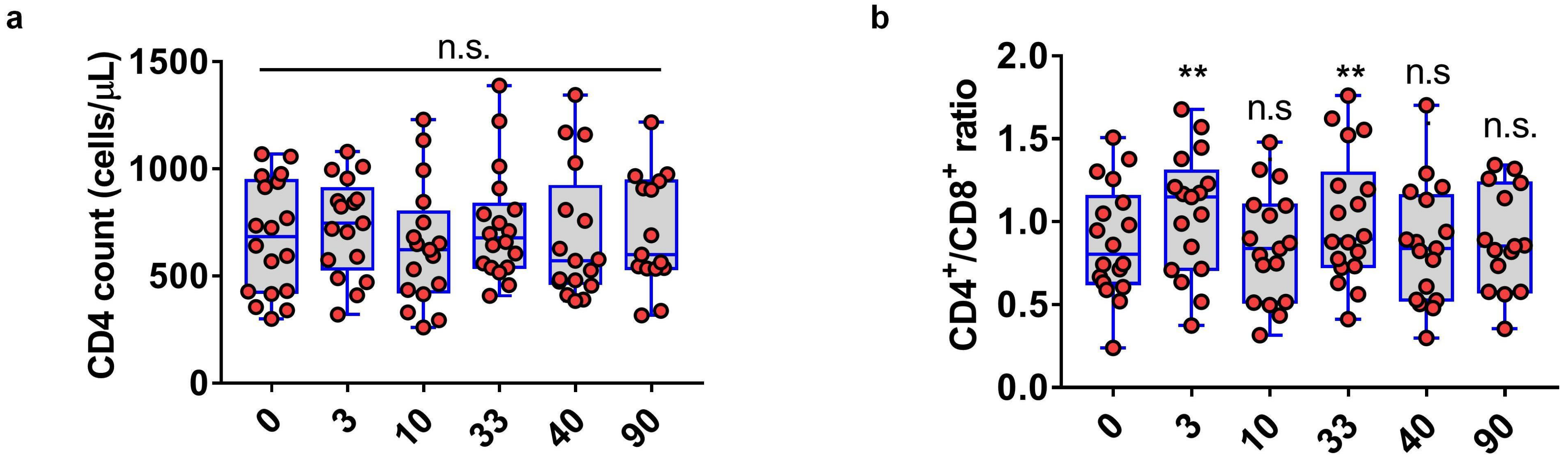

Supplementary Fig. S7

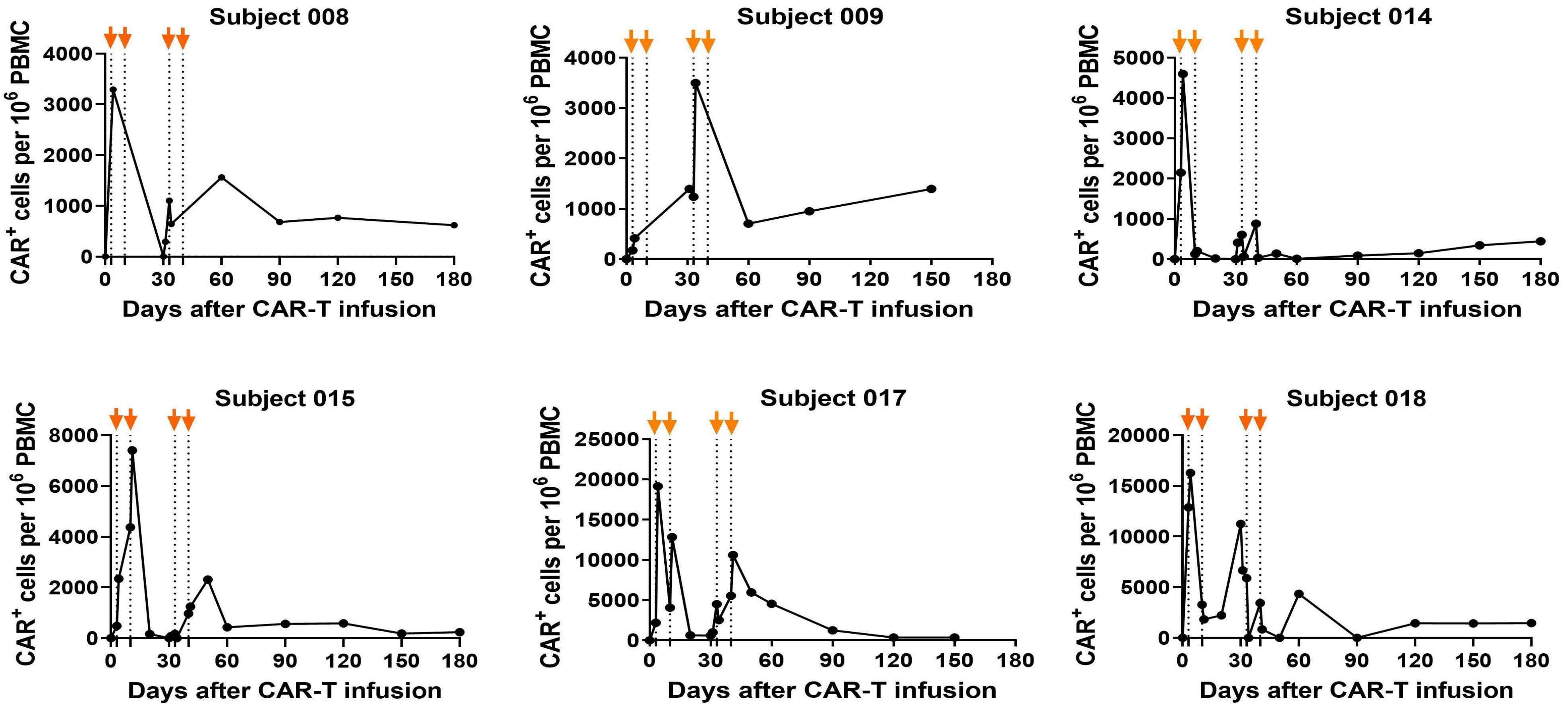

Supplemental Figure legends

Supplementary Fig. S1 The M10 cells showed decreased susceptibility to HIV-1 IIIB viruses compared to $CD8^+$ T cells.

Purified $CD4^+$ T cells, $CD8^+$ T cells, M31 $CD8^+$ CAR-T cells or M10 $CD8^+$ CAR-T cells were cocultured with HIV-1 IIIB viruses at an MOI of 0.2 and 2 respectively. The percentages of $p24^+$ cells were measured at Day 4 and Day 8 postinfection by flow cytometry after intracellular staining with an PE-conjugated p24-specific antibody (anti-p24-PE). $CD4^+$ T cells served as a positive control.

Fig. S2 M10 cells showed effective cytotoxic efficacy in vivo.

(A-B) Graphs presenting the growth of $gp145^-$ and $gp145^+$ cells in mice treated with untransduced T cells (UTD), M31 CAR-T cells and M10 CAR-T cells (n = 4 mice).

Fig. S3 The memory phenotypes were characterized for the M10 CAR-T products.

(A) Contour plots (n = 3) and (B) collated data (n = 8) are shown. Memory subsets were based on CD45RA and CD62L expression, and reported as terminally differentiated effector memory (T_{EMRA}), effector memory (T_{EM}), central memory (T_{CM}) and stem cell memory (T_{SCM}). Gates were set based on measurements of FMO controls.

Fig. S4 Favorable safety profile of M10 cells after infusion.

(A-F) Mean ± SEM of body temperature (A), pulse oxygen saturation (B), systolic and diastolic blood pressure (C-D), heart and respiratory rates (E-F) are shown at the indicated times after M10 cells infusion. (G) Concentrations of listed cytokines in serum obtained from participants at the indicated time points.

Fig. S5 Detection of anti-CAR antibodies in the serum of 12 participants that received infusions of M10 CAR-T cells.

Within the $CD3^+$ population, cells were analyzed for expression of CAR and IgG. The percent of CAR^+IgG^+ in the serum of the participants at baseline, one month post-treatment (M1), and three months (M3) post-treatment were analyzed by flow cytometry.

Fig. S6 CD4 counts and CD4/CD8 ratios of study participants.

(A) The changes in CD4 counts of all participants at indicated timepoints (n = 18). (B) The changes in the ratio of $CD4^+$ to $CD8^+$ T cells at indicated timepoints (n = 18).

Fig. S7 The in-vivo persistence and proliferation of M10 cells after infusion.

Measured CAR^+ cell counts (per million PBMCs) for the 6 enrolled participants are shown. The adoptive transfer of M10 CAR-T cells was performed on Day 0 and Day 30 followed by double administrations of chidamide shown as orange arrows in the panels.

Supplemental Table S1. The recipient-donor relationship, CAR-T cell dose, and CAR transduction efficiency.

Sub. ID	Allogenic donor	Dose (CAR^+ T cells/kg)	Course (First/ Second)	Total No. of infused cells, ×10^7	Percentage of $CD3^+$ cells	Percentage of $CD8^+$ T cells	Percentage of infused cells (CAR^+ $CXCR5^+$)	Percentage of infused cells (CAR^+ $CXCR5^-$)	Percentage of infused cells (CAR^- $CXCR5^+$)	No. of infused CAR^+ cells, ×10^7
001	Mother	1E6	First	19.3	99.3%	46.2%	2.6%	23.3%	4.4%	5.0
			Second	7.6	98.5%	42.5%	3.5%	62.6%	0.9%	5.0
002	Son	1E6	First	27.3	99.1%	61.5%	2.4%	15.9%	5.3%	5.0
			Second	20.1	98.7%	49.7%	3.0%	21.9%	4.3%	5.0
003	Father	1E6	First	40.7	98.6%	29.8%	2.8%	9.5%	15.0%	5.0
			Second	31.9	98.8%	14.7%	3.9%	10.2%	13.1%	4.5
004	Father	1E7	First	116.6	99.1%	24.4%	6.6%	41.7%	3.7%	50.0
			Second	101.0	98.7%	37.8%	1.4%	8.6%	6.4%	10.1
005	Daughter	1E7	First	73.4	98.9%	57.8%	12.4%	64.1%	1.7%	50.0
			Second	54.0	98.2%	65.5%	2.6%	23.0%	3.1%	13.8
006	Mother	1E7	First	95.3	99.2%	33.9%	14.4%	23.7%	3.9%	36.3
			Second	44.0	98.9%	47.5%	9.8%	35.3%	6.0%	19.8
007	Daughter	1E7	First	102.2	98.6%	69.8%	31.4%	17.5%	38.6%	50.0
			Second	69.2	99.3%	67.7%	31.4%	22.1%	15.9%	37.0
008	Father	1E7	First	88.0	98.1%	21.7%	7.2%	29.4%	14.7%	32.2
			Second	44.8	97.2%	56.6%	25.4%	19.2%	18.8%	20.0
009	Mother	1E7	First	83.3	99.6%	55.5%	2.9%	9.1%	13.9%	10.0
			Second	74.6	98.2%	45.7%	6.1%	25.4%	12.4%	23.5
010	Mother	1E7	First	37.0	98.6%	35.0%	8.4%	34.7%	6.15%	15.9
			Second	34.0	99.2%	24.0%	6.5%	62.1%	1.6%	23.3
011	Sister	1E7	First	74.6	99.1%	73.4%	25.0%	42.0%	5.64%	50.0
			Second	89.8	99.5%	60.1%	29.7%	26.0%	12.5%	50.0

012	Father	1E7	First	96.1	98.0%	55.1%	24.1%	24.5%	15.3%	46.7
			Second	117.4	99.0%	28.9%	24.6%	18.0%	18.2%	50.0
013	Father	1E7	First	50.0	98.2%	48.4%	16.7%	35.8%	7.69%	26.3
			Second	84.5	97.8%	48.8%	15.2%	23.1%	13.5%	32.4
014	Mother	1E7	First	58.0	98.7%	37.9%	51.8%	29.3%	7.7%	47.0
			Second	88.0	98.5%	44.0%	32.7%	20.6%	20.8%	46.9
015	Mother	1E7	First	42.3	98.7%	30.6%	14.8%	21.8%	12.3%	15.5
			Second	80.8	99.3%	25.5%	31.2%	8.4%	5.9%	32.0
016	Mother	1E7	First	61.0	99.0%	15.3%	8.4%	17.5%	6.2%	15.8
			Second	42.5	99.5%	33.3%	19.5%	21.5%	9.7%	17.0
017	Father	1E7	First	65.0	98.1%	21.2%	6.0%	17.7%	7.1%	15.4
			Second	61.8	98.6%	10.1%	5.7%	15.7%	9.4%	13.2
018	Father	1E7	First	98.0	99.6%	50.6%	7.4%	18.5%	6.5%	25.4
			Second	71.3	98.7%	71.0%	13.9%	31.7%	8.4%	32.5

Supplemental Table S2. Protein sequence information of CAR constructs.

Peptide	Detailed sequence information
m36.4	QVQLVQSGGGLVQPGGSLRLSCAASAFDFSDYEMSWVREAPGKGLE<br>WIGEINDSGNTIYNPSLKSRVTISRDNSKNTLYLQMNTLRAEDTAIYY<br>CAIYGGNSGGEYWGQGTLVTVSS
mD1.22	KKVVYGKKGDTVELTCTASQKKNIQFHWKNSNQIKILGNQGSFLTK<br>GPSKLNDRVDSRRSLWDQGNFPLIIKNLKPEDSDTYICEVEDQKEEV<br>QLVVVG
10E8scFv-Fc	MGWSCIILFLVATATTGSYELTQETGVSVALGRTVTITCRGDSLRSHY<br>ASWYQKKPGQAPILLFYGKNNRPSGVPDRFSGSASGNRASLTISGAQ<br>AEDDAEYYCSSRDKSGSRLSVFGGGTKLTVLGGGGSGGGGSGGGGS<br>EVQLVESGGGLVKPGGSLRLSCSASGFDFDNAWMTWVRQPPGKGL<br>EWVGRITGPGEGWSVDYAAPVEGRFTISRLNSINFLYLEMNNLRMED<br>SGLYFCARTGKYYDFWSGYPPGEEYFQDWGRGTLVTVSSEPKSCDK<br>THTCPPCPAPELLGGPSVFLFPPKPKDTLMISRTPEVTCVVVDVSHED<br>PEVKFNWYVDGVEVHNAKTKPREEQYNSTYRVVSVLTVLHQDWLN<br>GKEYKCKVSNKALPLPEEKTISKAKGQPREPQVYTLPPSRDELTKNQ<br>VSLTCLVKGFYPSDIAVEWESNGQPENNYKTTPPVLDSDGSFFLYSK<br>LTVDKSRWQQGNVFSCSVMHEALHNHYTQKSLSLSPGK
